# Atomic Group Decomposition of Charge Transfer Excitation Global Indexes

**DOI:** 10.1021/acs.jpca.2c04607

**Published:** 2022-09-02

**Authors:** Carlo Gatti, Yann Danten, Christine Frayret

**Affiliations:** † CNR Istituto di Scienze e Tecnologie Chimiche “Giulio Natta”, CNR SCITEC, Sede Via C. Golgi, 19, 20133 Milano, Italy; ‡ Istituto Lombardo, Accademia di Scienze e Lettere, via Brera 76, 20121 Milano, Italy; § Institut des Sciences Moléculaires, UMR CNRS 5255, Université de Bordeaux, 351 Cours de la Libération, 33405 Talence, France; ∥ Laboratoire de Réactivité et Chimie des Solides (LRCS), UMR CNRS 7314, Université de Picardie Jules Verne, Hub de l’Energie, 15 Rue Baudelocque, 80000 Amiens Cedex, France; ⊥ Réseau sur le Stockage Electrochimique de l’Energie (RS2E), FR CNRS 3459, 80039 Amiens Cedex, France

## Abstract

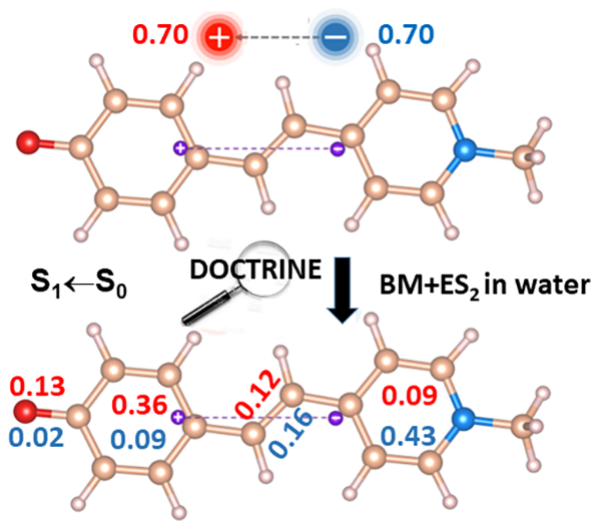

A model for decomposing the Le Bahers, Adamo, and Ciofini Charge Transfer (CT) Excitations global indexes (J. Chem. Theory Comput.2011, 7, 2498−25062660662410.1021/ct200308m) into molecular subdomains contributions is presented and a software, DOCTRINE (atomic group Decomposition Of the Charge TRansfer INdExes) for the implementation of this novel model has been coded. Although our method applies to any fuzzy or to any disjoint exhaustive partitioning of the real space, it is here applied using a definition of chemically relevant molecular subdomains based on the Atoms in Molecules Bader basins. This choice has the relevant advantage of associating *intra* or *inter* subdomain contributions to rigorously defined quantum objects, yet bearing a clear chemical meaning. Our method allows for a quantitative evaluation of the subdomain contributions to the charge transfer, the charge transfer excitation length and the dipole moment change upon excitation. All these global indexes may be obtained either from the electron density increment or the electron density depletion upon excitation. However, the subdomain contributions obtained from the two distributions generally differ, therefore allowing to distinguish whether the contribution to a given property of a given subdomain is dominated by one of the two distributions or if both are playing a significant role. As a *toy* system for the first application of our model, a typical [D−π–A, π = conjugated bridge] compound belonging to the merocyanine dyes family is selected, and the first four excited states of this compound in a strongly polar protic solvent and in a weakly polar solvent are thoroughly investigated.

## Introduction

Computation of the electronic structure of excited states has made enormous strides over the past decade, due to the combined effect of the continuous, fast increase of computer power and even more so of the massive investment that has been made to develop new and ever more powerful electronic structure methods.^1−^^4^ The ability of performing careful computations of electron transitions and of evaluating the excited state wave functions and properties of larger and larger molecular systems has also stimulated significant efforts to devise methods able to analyze their excited state electronic structure. Often, the latter becomes increasingly complex with increasing size of the systems and new qualitative physics behavior also emerges. Methods for automatizing the excited state analysis, supported by visualization techniques^5−7^ and aimed at providing rigorous and reproducible descriptors to measure charge transfer (CT),^8−13^ double excitation character,^7,14−16^ entanglement,^17−20^ and, more generally, at disclosing phenomena that are hidden in the standard molecular orbital (MO) picture,^11,21,22^ have been presented. TheoDORE^21,22^ is one of the most successful and user-friendly available method and package in this area, being interfaced to ten different quantum chemistry codes and to a range of excited-state methods implemented therein. Three powerful functionalities of TheoDORE have become particularly popular, namely a “fragment-based analysis for assigning state character, the computation of exciton sizes for measuring charge transfer, and the natural transition orbitals used not only for visualization but also for quantifying multiconfigurational character”.^21^

In the present manuscript, we introduce yet another new method able to provide a rigorous decomposition of CT excitation descriptors (also called CT global indexes) into atomic group (or molecular subdomain) contributions. The method represents a generalization to molecular subdomains of the CT global indexes model developed some years ago by Le Bahers, Adamo, and Ciofini (hereafter LBAC model).^9^ In such a model, a measure of the length of a CT excitation is defined on the basis of the sole knowledge of the system’s ground and excited state electron densities (EDs). Within the LBAC method, the barycenters or centroids of the ED depletion and ED enrichment regions upon electron transition are computed, and the CT length is taken as the distance between these centroids. Then the transferred charge is obtained by integrating either the ED depletion or the ED enrichment distribution over the whole molecular space. From the CT length and the transferred charge, the change of the dipole moment upon electron transition is computed and compared with that obtained from the computed *ab initio* ground state and excited state wave functions, so enabling to check the accuracy of the adopted integration procedures. Our model development allows for decomposing the key quantities of the LBAC model into molecular moieties contributions with the moieties being defined in terms of a fuzzy or a disjoint exhaustive partitioning of the real space. The choice of subdomains is arbitrary, yet chemically meaningful moieties should be preferentially used to gain chemical insight into the CT process. Our method bears some resemblance to the fragment-based analysis in TheoDORE^21,22^ that has proved to be particularly useful for singling out the system fragments that mostly contribute to a given excitation and for detecting in which portions of a molecule CT occurs. Yet, the TheoDORE method^21^ strongly differs from our presented approach being based on the concept of a correlated electron pair and on the use of wave function for the electron–hole pair rather than on the N-electron wave functions of the ground and excited states. In practice, TheoDORE bases its CT analysis on the one-electron transition density matrix, while both the LBAC model^9^ and our generalization of the LBAC model to subdomains uses the *rearrangement* ED, which is the difference of the excited and GS EDs. In TheoDORE, charge transfer “numbers”^21^ within a fragment and between fragments are customarily calculated through population analysis schemes and may be somewhat or largely basis set and computational method dependent, while in our model intra- and interfragment contributions are evaluated in the real space of subdomains basins and are thus much less basis set and method dependent, provided both of them are of a sufficient quality.

Using the length and magnitude of charge transferred, the LBAC model was devised to screen on a qualitative basis push–pull compounds belonging to diverse chemical families,^9,10^ thereby providing experimental chemists with useful insights to design push–pull compounds with targeted properties. Push and pull systems, consisting of an electron donor (D) and of a covalently connected electron withdrawing (A) group have been largely investigated owing to their intense, solvatochromic, optical transitions.^9,10,22−26^ However, the simple mechanism of the formation of an excited state where an electron is transferred from the D to the A to form a [D^+^A^–^]* excited state is just an ideal situation.^27^ Excitations may, in reality, be local or delocalized in character, they may not take place necessarily from the D to the A, and besides that, the effective transferred charge may be much lower than the ideal value of one for single-electron excitation processes.^21,26−28^

Photoinduced electron-transfer (PET) is a key mechanism of various chemical, physical, or biological processes, all having intensively scrutinized applications areas, including light-to-chemical energy conversion, molecular photoelectronics, photocatalysis, or photosynthesis. The ability of tuning and/or improving charge transfer and of identifying the actors playing the major role into the electron-transfer mechanisms is clearly a prerequisite for designing optimized systems.

Extension of the LBAC model to a subdomain representation, enabling us to distinguish and quantify the local intrasubdomain effects from the synergic or anti-synergic coupled effects of all subdomain pairs on the global CT indexes, may hopefully provide further precious insight on the PET processes.

**Scheme 1:**
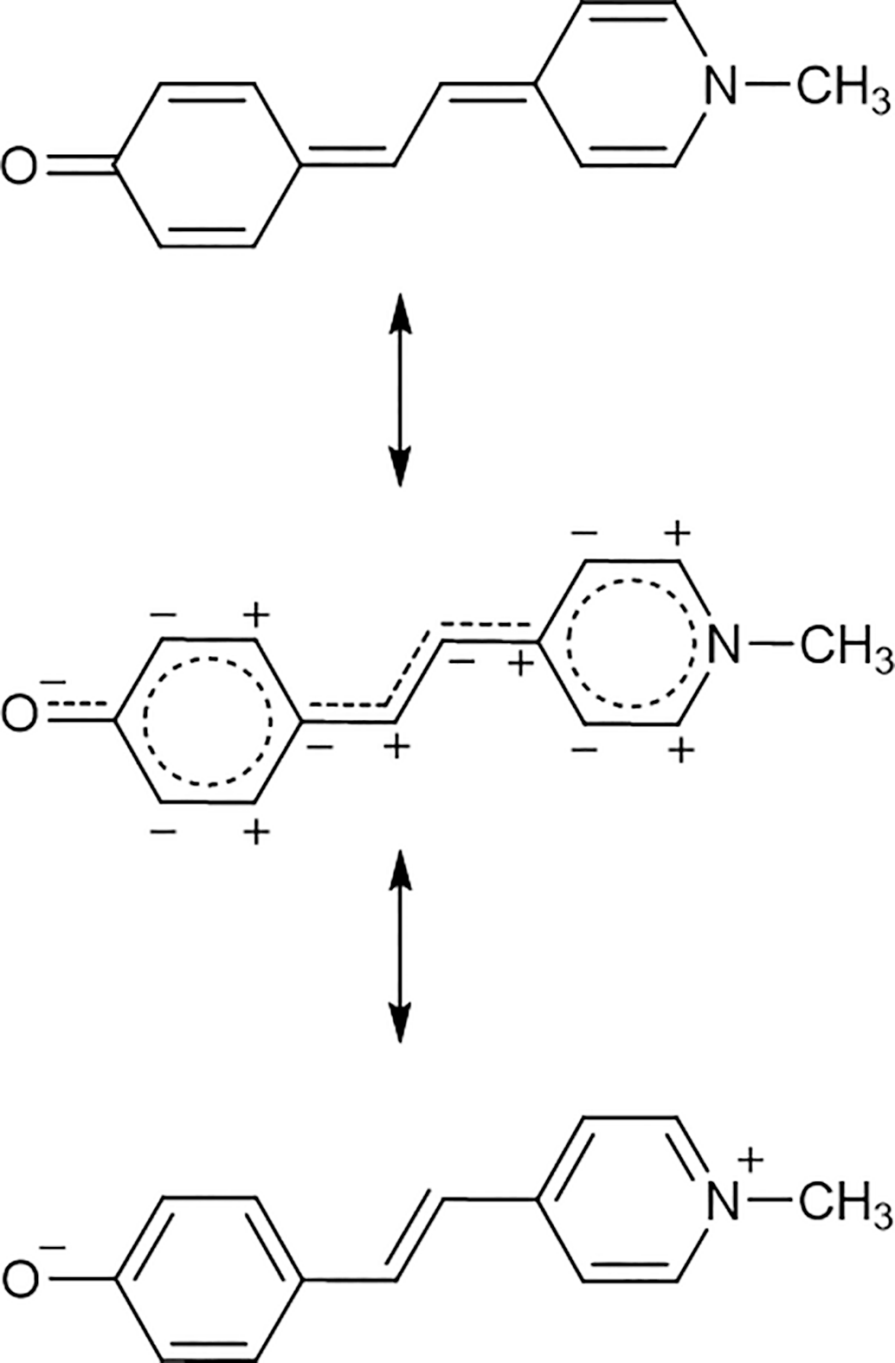
Limiting Resonance Forms of the Brooker’s Merocyanine (BM) Molecule: Bond-Alternated Polyene-like (Top); Non-bond-alternated Polymethine-like (Middle); Bond-Alternated, Fully Reversed Zwitterionic State (Bottom)

As a “toy” system for the first application of our method, we selected a typical [D−π–A, π = conjugated bridge] compound belonging to the merocyanine dyes family, namely the 1-methyl-4-[(4-oxocyclohexadienylidene)ethylidene]-1,4-dihydropyridine, also called Brooker’s Merocyanine (BM), taken in its trans-deprotonated form (Scheme 1). In this molecule, the electron-donating D (CH_3_N) and electron acceptor A (CO) groups are separated by a conjugated system, enabling the electron drift between these two components. Its electronic structure may be envisaged as a *resonance hybrid* between two limiting structures, a *noncharge-separated* one (covalent, strongly bond-alternated, neutral polyene-like) and a *charge-separated* structure (bond-alternated, zwitterionic form) (Scheme 1).^30^ Yet, an intermediate form, called the cyanine-like limit or polymethine-like (BM)_pm_ (nonalternated, Scheme 1) state, which lies in between the quinoid and benzenoid limits and has intermediate charge-transfer states, has been often invoked to interpret the various BM spectroscopic properties.^31,32^ Quite interestingly, BM is well-known for being able to finely tune its chemical bonding features concomitantly with the electronic CT arising across the molecule. Under photoexcitation involving the S_0_–S_1_ transition, the compound—which is sensitive to solvatochromic effects—is considered to typically switch its π-electronic structure from an aromatic benzenoid (BM)_b_ to a quinonoid pro-aromatic (BM)_q_ structure in protic solvents, the opposite being generally true in apolar solvents.^33^

We have recently performed a systematic, comprehensive Density Functional Theory (DFT) and Time Dependent Density Functional Theory (TD-DFT) study of the photoinduced CT processs in the BM compound, as a function of the various BM excited states and of the adopted solvent model.^33^ In this work, the trans-deprotonated form of the BM molecule was considered in various solvent media characterized by a wide spectrum of dielectric constants and by making use of either only an implicit (SMD) solvent model or the same SMD model but with a further inclusion of explicit solvent molecules.

The aim of this recent study was to exploit the possibility to reach a suited manipulation of the CT process thanks to the quite different nature, in a given solvent, of the first four BM excited states, and to the significant alteration of the excited states nature that may be induced by choosing among 24 different solvent models. Electronic transitions were first characterized in terms of LBAC global indexes (the effective amount of transferred charge *q*_*CT*_ upon excitation, the CT excitation length, *D*_*CT*_, and the resulting change in the magnitude of the molecular dipole, |Δ**μ**_CT_|). All these global indexes were calculated through our implementation of the standard LBAC model. Additionally, LBAC indexes were also put in relation with the evolution of local features that characterize—upon vertical excitations—either the chemical bonds or the electron delocalization in the various BM moieties.^33^

In the present manuscript, we use, instead, a few representative cases from that study to explore the further chemical and physical insight that an atomic group decomposition of LBAC CT indexes may hopefully provide.

## Theory and Computational Details

The LBAC model^9^ is briefly reviewed below, and its new atomic group decomposition version is outlined, along with details on its practical implementation. Computational details for the application of the developed method to the BM system are then presented.

### A Qualitative Index of the Spatial Extent in Charge-Transfer (CT) Excitations: The LBAC Model

Named as *ρ*_*GS*_ and ρ_*EX*, *n*_ the electronic densities of the ground state (GS) and of the vertical excited state *n*, respectively, the ED rearrangement due to the electronic transition S_*n*_ ← S_0_ is given by
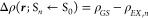
1where Δρ denotes a local increment (Δρ < 0) or a local depletion (Δρ > 0) of the ED upon electronic transition and where Δρ integrates to zero over the whole space *R*^3^ (for the sake of simplicity, Δρ(***r***; S_*n*_ ← S_0_) is hereafter simply written as Δρ(***r***)). By defining ρ^+^(***r***) and ρ^–^(***r***) as being equal to |Δρ(***r***) | if Δρ(***r***) is, respectively, greater or smaller than zero, and equal to zero if Δρ(***r***) is, respectively, smaller or greater than zero, i.e.:
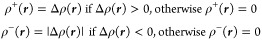
2it is easy to obtain the amount of transferred charge *q*_*CT*_ upon excitation as the following quantity:
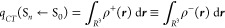
3

*q*_*CT*_ will range between 0 and 1 for a one-electron excitation, being close to 1 for a nearly ideal one-electron transfer and even much less than so in real situations. The centroids or the locations of the poles of the positive and negative transferred charge are given by
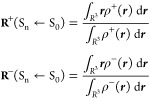
4and, accordingly, a measure of the CT excitation length, *D*_*CT*_, is obtained from
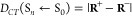
5

The norm of the dipole moment change between the ground and the excited state, **μ**_*CT*_, is calculated as
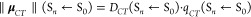
6and the, hopefully negligible, departure of this norm from the difference between the *ab initio* dipole moment magnitudes computed for the ground and the excited state *n* gives an estimate of the integration accuracy in eqs 3 and 4 (see above).

It is important to note that in eq 1 an opposite sign convention relative to the original LBAC model was adopted. Both sign conventions are possible and correct. Our specific choice allows for associating positive Δρ values to the regions that upon transition to the excited state decrease their electron concentration (hence becoming *positively charged* relative to the GS) and negative Δρ values to the regions that increase their electron concentration (hence becoming *negatively charged* relative to the GS).

### Atomic Group Decomposition of the LBAC Model CT Indexes

By assuming a fuzzy or a disjoint exhaustive space partitioning of *R*^3^ in subdomains Ω, eq 3 may be written as
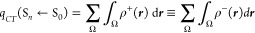
7enabling to envisage *q*_*CT*_ as caused by a sum of subdomains contributions. Notice that the equivalence of the sums of subdomain contributions calculated by integrating either ρ^+^(***r***) or ρ^–^(***r***) over the whole set of Ω does not hold true for the separate subdomain contributions. In general, ∫_Ω_ρ^+^(***r***) d***r*** ≠ ∫_Ω_ρ^–^(***r***) d***r*** and the following subdomain quantities may be defined as follows:
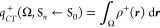
8a
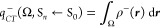
8b
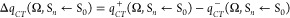
8c

Equations 8a–8c tell us three important facts, namely that a subdomain (i) may have regions contributing to the positive pole and regions contributing to the negative pole of the transferred charge, (ii) that such contributions may be different (even largely) in magnitude, and (iii) that it may be convenient to define also their difference Δ*q*_*CT*_(Ω) to appreciate whether a subdomain is more responsible of creating one or the other of the two poles. Note also that ∑_Ω_Δ*q*_*CT*_(Ω, S_*n*_ ← S_0_) = 0 and that the following inequalities hold
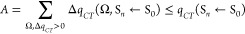
9a
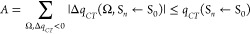
9bwhere the equal sign in eqs 9a and 9b is only achieved in the very unlike situation of any subdomain having either only positive or only negative Δρ(***r***) values.

Likewise *q*_*CT*_, also *D*_*CT*_ may be conveniently written in terms of subdomain contributions,
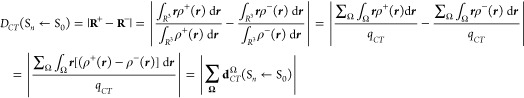
10where **d**_*CT*_^Ω^, different from *D*_*CT*_, is a 3-component vector. Hence, we introduce the corresponding vector for the whole system, **d**_CT_, whose components are given by a sum over the corresponding subdomain components:
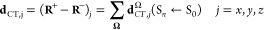
11while
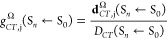
12is a dimensionless quantity providing a measure of the **d**_*CT*, *j*_^Ω^(S_*n*_ ← S_0_) length relative to the *D*_*CT*_ length. A negative sign of *g*_*CT*, *j*_^Ω^ means that **d**_*CT*, *j*_^Ω^ is oppositely directed to (**R**^+^ – **R**^–^)_*j*_. Also useful is to introduce the direction cosines of (**R**^+^ – **R**^–^), α_*j*_ (*j* = *x*, *y*, *z*), i.e.:
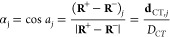
13These express the different degree of alignment of the three components of **d**_CT_ to (**R**^+^ – **R**^–^) and where *a*_*j*_ is the angle between the *j* axis and the (**R**^+^ – **R**^–^) vector.

As indicated in eq 6, the norm of the dipole moment change between the ground and the excited state, ∥**μ**_*CT*_∥(S_*n*_ ← S_0_), is given by the product of *D*_*CT*_ and *q*_*CT*_ and its decomposition in subdomain contributions is not straightforward for two main reasons. First, one has to introduce a separate decomposition for each dipole moment change component:
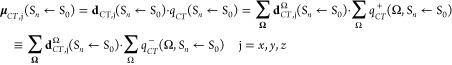
14and second, the product in eq 14 unavoidably contains mixed terms involving pairs of subdomains. Although a formal single subdomain decomposition of each **μ**_*CT*, *j*_ would be possible, e.g. by assuming to assign to each subdomain half of its subdomain pairs contributions, this partitioning remains arbitrary, and we thus prefer to avoid in this manuscript any subdomain decomposition based on eq 14. However, the properties of the not symmetric square matrix *M*^*j*,+^ having as diagonal elements the single subdomain contributions **d**_*CT*, *j*_^Ω^·*q*_*CT*_^+^(Ω) and as out-of-diagonal elements the mixed terms **d**_*CT*, *j*_^Ω^·*q*_*CT*_^+^(Ω ′) are of some interest and are worth investigating. For instance, each **μ**_*CT*, *j*_ may be easily decomposed into an intrasubdomains contribution, **μ**_*CT*, *j*_^*intra*,+^, and in an intersubdomains counterpart, **μ**_*CT*, *j*_^*inter*,+^, as follows:
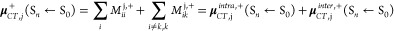
15The *M*^*j*,+^ matrix is dimensioned *nsub* times *nsub* where *nsub* is the number of considered subdomains. Clearly an analogous *M*^*j*,–^ matrix may also be defined, having as diagonal elements the single subdomain contributions **d**_*CT*, *j*_^Ω^·*q*_*CT*_^–^(Ω) and as out-of-diagonal elements the mixed terms **d**_*CT*, *j*_^Ω^·*q*_*CT*_^–^(Ω ′). Both matrices *M*^*j*,+^ and *M*^*j*,–^ have the property to reproduce the ∥**μ**_*CT*_∥ value from either their **μ**_*CT*, *j*_^+^ or **μ**_*CT*, *j*_^–^ vector components, or equivalently by summing up either all the *M*^*j*,+^ or all the *M*^*j*,–^ matrix elements (eq 15). Yet, the corresponding *M*_*ik*_^*j*,+^and *M*_*ik*_^*j*,–^matrix elements are generally different from each other. The **μ**_*CT*, *j*_ decomposition afforded by eq 15 gives a measure of the extent of subdomain interdependency in determining the variation of the *j* component of the dipole moment upon excitation. The diagonal, *M*_*ii*_^*j*,+^, and the out of diagonal elements, *M*_*ik*_^*j*,+^ and *M*_*ki*_^*j*,+^ with *M*_*ik*_^*j*,+^ ≠ *M*_*ki*_^*j*,+^ in general), represent the subdomain Ω_*i*_ internal contribution and the subdomain Ω_*i*_Ω_*k*_ pair contributions, respectively.

A much simpler subdomain decomposition of ∥**μ**_*CT*_∥ may be realized if only the dependence of ∥**μ**_*CT*_∥ on *q*_*CT*_ is taken into account. In such a case, a quite simple expression results:
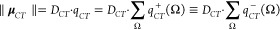
16a
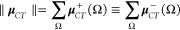
16bwhere one obtains, as it should be, a single value for ∥**μ**_*CT*_∥ but in terms of two different subdomain decompositions, one based on *q*_*CT*_^+^(Ω) and the other on *q*_*CT*_^–^(Ω). It is of some interest to compare these two alternative subdomain decompositions of ∥**μ**_*CT*_∥, one emphasizing the role of *q*_*CT*_^+^(Ω) and the other that of *q*_*CT*_^–^(Ω). Both decompositions clearly have a chemical significance.

### Implementing Atomic Group Decomposition of the LBAC Model CT Indexes

As stated earlier, eqs 7–16 hold true regardless if the fuzzy boundary or disjoint exhaustive space partitioning schemes are adopted. However, the use of the Quantum Theory of Atoms in Molecules (QTAIM)^34^ zero flux condition for defining the subdomains Ω, i.e.,
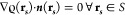
where **r**_*s*_ is any point on the subdomain surface boundary *S* and ***n***(**r**_*s*_) is the normal to the surface at **r**_*s*_, enables us to associate the CT indexes subdomain contributions to atoms or groups of atoms rigorously defined through quantum mechanics.^34^Eqs 7–16 are all related to two-state quantities. The QTAIM space partitioning, as for any other not purely geometrical partitioning, is instead a function of the molecular state, so one has to make an assumption about which of the two involved states is selected as a reference for the atomic and atomic groups space partitioning in the eqs 7–16. In order to have a common reference for the series of the excited states of a molecule, we have always selected the space partitioning associated with the molecule in the GS. Note that, analogously to the LBAC model, only vertical excitations can be considered in the subdomain version of this model, so one expects that the change in the subdomain boundaries upon excitation is (highly) dampened relative to the case of adiabatic electronic transitions. In other words, using either the ground state or the excited state atomic boundaries should not dramatically change the picture of the CT global index subdivisions in atomic group contributions.

Based on the just illustrated premises, we have written a code, DOCTRINE^35^ (atomic group Decomposition Of the Charge TRansfer INdExes), that, as a first step, calculates all the QTAIM atomic basin contributions to the CT indexes. In practice, at this stage, Ω is any atomic basin of the molecule in the eq 7-16. DOCTRINE makes use of the wave function files, in .wfn format, and obtained from the ab initio GAUSSIAN-16 code,^36^ of the ground and of the excited state *n* of the molecule being investigated, along with the file (in .sur format) containing the surface boundary information on all QTAIM atomic basin Ω of the molecule in the ground state. The data in the .sur file are computed by a previous PROMEGA calculation (PROMEGA is one of the codes of Bader’s AIMPAC95 package)^37^ that evaluates the boundaries of all atomic basins in the GS molecule. Integral properties of the QTAIM basins may also be calculated at this stage if of interest for relating them to the computed CT indexes^33^ and to their atomic group decomposition. Clearly, in such a case, the atomic boundaries and the integral properties of the excited state need also to be computed to evaluate the integral properties changes upon electron transition. DOCTRINE evaluates ∥**μ**_*CT*_∥(S_*n*_ ← S_0_) from eq 6 (or through eq 14 or eq 16). Comparison of the reconstructed value of ∥**μ**_*CT*_∥(S_*n*_ ← S_0_) from its atomic basin Ω contributions with the value computed by the GAUSSIAN 16 code permits to evaluate the accuracy of the numerical integration. Since this is performed for all QTAIM atoms in spherical coordinates and on an atomic centered grid, the resulting accuracy is noteworthy, provided a suitable number of angular and radial points is used in the Gaussian quadrature integration procedure (see Computational Details). Further checks of integration accuracy are provided by comparing quantities that should be ideally equivalent, such as the ∥**μ**_*CT*_∥(S_*n*_ ← S_0_) values obtained from eq 6 (or through eq 14 or eq 16), either using *q*_*CT*_^+^ (Ω) or *q*_*CT*_^–^ (Ω) data or the *q*_*CT*_ values obtained from eq 7, either using ρ^+^ or ρ^–^ distributions.

Once the QTAIM atomic basin Ω contributions to the CT indexes have been calculated, DOCTRINE combines and gathers them into the selected *nsub* atomic group contributions, each atomic group being composed by a suitably selected disjoint subset of the atoms of the investigated molecule. Clearly, the so defined *nsub* molecular subdomains need to collectively include all atoms of the molecule.

### Application of the Developed Method to the BM System

For this study of the atomic groups decomposition of the LBAC global indexes, we have used the GS and the first four excited states of the BM molecule in CCl_4_ and in water, as representative examples. CCl_4_ is a weak polar nonhydroxyl solvent (dielectric constant ε = 2.23), while water is a strong polar protic solvent (ε = 78.4). In the case of water, two explicit solvent (ES) molecules have been considered, since the formation of hydrogen bonds (HBs) between the carbonyl end of the BM molecule and the hydrogen atoms of water solvent molecules has been suggested to present strong involvement in the electron transition properties. Furthermore, the crystallized BM compound includes structural solvent molecules.^38,39^ This explicit solvent model for BM in water is hereafter referred to as BM + ES_2._ For both solvent cases, the following four BM moieties were considered in our analysis: the carbonyl oxygen atom, the 6-Carbon Membered Ring plus its four linked H atoms, the two C atoms of the Central Bridge plus their two linked H atoms and the MethylPyridine ring along with its linked 4 H atoms. In the following, these four BM moieties (≡ BM Ω subdomains) will be called as O(CO), 6CMR, CB and MePy, respectively. The .wfn files for the two ground states and eight excited states investigated in the present study have been taken from our previous work on the BM molecule excitations.^33^ Quantum chemical calculations were carried out through the Gaussian 16 software package and using DFT and TD-DFT procedures. Geometry optimization [S_0_ or singlet ground state (GS)] and TD-DFT treatment for the vertical excited states (S_*n*_, *n* = 1–4) were all performed using the long-range corrected (LRC) Coulomb attenuated B3LYP *xc* functional CAM-B3LYP^40^ with the cc-pVDZ basis set and by including solvent effects through the implicit SMD solvation model.^41^

In the case of BM + ES_2_ for the water model, besides considering the implicit dielectric effect of solvent through SMD, two explicit water molecules were initially placed in the neighborhood of the C=O group and left totally free to geometrically relax during the geometry optimization step. The local energy minimum nature of the calculated structures was confirmed from their (harmonic) vibrational analysis implemented in the Gaussian 16 software package (no imaginary frequency).

The global CT indexes, along with their atomic group decomposition, have been obtained from the DOCTRINE code (see previous paragraph). For the BM + ES_2_ in water system, the adopted .wfn files for both the GS and the excited states were those obtained by restraining excitations and charge transfer to the BM molecule only (i.e., the wave functions were generated by setting zero charge on solvent molecules while using the basis set of the complete system). We had however to verify that no CT occurs to the explicit solvent molecules also when a QTAIM space partitioning is adopted. Indeed, the QTAIM net charge on the two explicit solvent water molecules has been always found to be below ±5 × 10^–3^ electrons for all investigated cases. These results enable us to restrain the decomposition of global CT indexes to contributions from subdomains formed by atoms of the BM molecule only also for the BM + ES_2_ in water system.

Atomic boundaries for the ground states were determined by PROMEGA code using typically 6144 angular points (96 and 64, for φ and θ, respectively). Evaluation by the DOCTRINE code of the atomic contributions to the global CT indexes used 200 radial points in the Gaussian quadrature integration procedure outside the so-called beta sphere and the same angular points of the previous determination of atomic boundaries by the PROMEGA code. The evaluation of atomic contributions to the global CT indexes is about 2 orders of magnitude faster than the atomic boundaries determination, and it is therefore very fast (typically 5–10 min for a system like BM, on a medium sized cluster of workstations). The CPU more demanding step, *i.e*., the atomic boundaries determination, needs however to be performed only for the ground state of a system, regardless of the number of its investigated excited states.

## Results and Discussion

### Accuracy of Global and Atomic Group CT Indexes

Table 1 reports the values of Δ∥**μ**_*CT*_∥ = ∥**μ**_*CT*_∥_DOCTRINE code_ – ∥**μ**_*CT*_∥_Gaussian 16 code_ for all investigated excitations. Δ∥**μ**_*CT*_∥ is the difference of the ∥**μ**_*CT*_∥ value obtained from the separate atomic or atomic group contributions to ∥**μ**_*CT*_∥ evaluated by the DOCTRINE code and that calculated directly by the Gaussian-16 code. The largest Δ∥**μ**_*CT*_∥ difference reported in Table 1 is as small as −0.173 D (D = Debye), relative to a dipole moment larger than 20 D, for the S_3_ ← S_0_ excitation of BM + ES_2_ in water. Typically, Δ∥**μ**_*CT*_∥ values are found to be one order or even 2 orders of magnitude smaller. Table 1 also lists the amounts of transferred charge *q*_*CT*_ upon excitation calculated by integrating either ρ^+^(***r***) or ρ^–^(***r***) over the whole set of atomic or atomic groups subdomains of the BM molecule, for all studied excitations. The differences between the *q*_*CT*_ values obtained using one or the other of the two densities, also reported in Table 1, provide a faithful indication of the noticeable integration accuracy of the *q*_*CT*_ values. Such differences never exceed 0.006 e^–^ and in most cases are even 1 to 2 orders of magnitude smaller.

**Table 1:** Accuracy of the Evaluation of the Norm of the Dipole Moment Change between the Ground and the Excited State *n,* ∥**μ**_*CT*_∥ (S_*n*_ ← S_0_), and of the Transferred Charge *q*_*CT*_ upon Excitation, Using the Atomic Group Subdomains Decomposition of the Global CT Indexes, for All the Investigated BM Excitations

state	∥**μ**_*CT*_∥, *D*	Δ∥**μ**_*CT*_∥, *D*^a^	*q*_*CT*_^+^ b	*q*_*CT*_^–^ b	*q*_*CT*_^+^ – *q*_*CT*_^–^
BM + ES_2_ in water					
S_1_ ← S_0_	12.958	–0.021	0.7000	0.7005	–0.0004
S_2_ ← S_0_	15.013	–0.035	0.7610	0.7616	–0.0005
S_3_ ← S_0_	20.835	–0.173	1.0248	1.0302	–0.0054
S_4_ ← S_0_	3.399	0.010	0.5134	0.5130	0.0004
BM in carbon tetrachloride					
S_1_ ← S_0_	0.704	0.050	0.3704	0.3704	0.0000
S_2_ ← S_0_	9.676	0.003	0.8604	0.8603	0.0001
S_3_ ← S_0_	10.049	–0.004	0.8138	0.8138	0.0001
S_4_ ← S_0_	4.663	0.029	0.5566	0.5566	–0.0001

a Dipole moments in Debye (D). Δ∥**μ**_*CT*_∥ = ∥**μ**_*CT*_∥_DOCTRINE_ – ∥**μ**_*CT*_∥_Gaussian 16_ is the difference between the ∥**μ**_*CT*_∥ value (column 2 in the table) obtained from the separate atomic or atomic group subdomains contributions to ∥**μ**_*CT*_∥ evaluated by the DOCTRINE code and that calculated directly by the Gaussian-16 code.

b *q*_*CT*_^+^ and *q*_*CT*_^–^ are the values of the transferred charge *q*_*CT*_ upon excitation calculated by integrating ρ^+^(***r***) or ρ^–^(***r***), respectively, over the whole set of atomic or atomic group subdomains of the BM molecule.

### Global CT Indexes and Their Atomic Group Subdomain Decomposition

**Figure 1:**
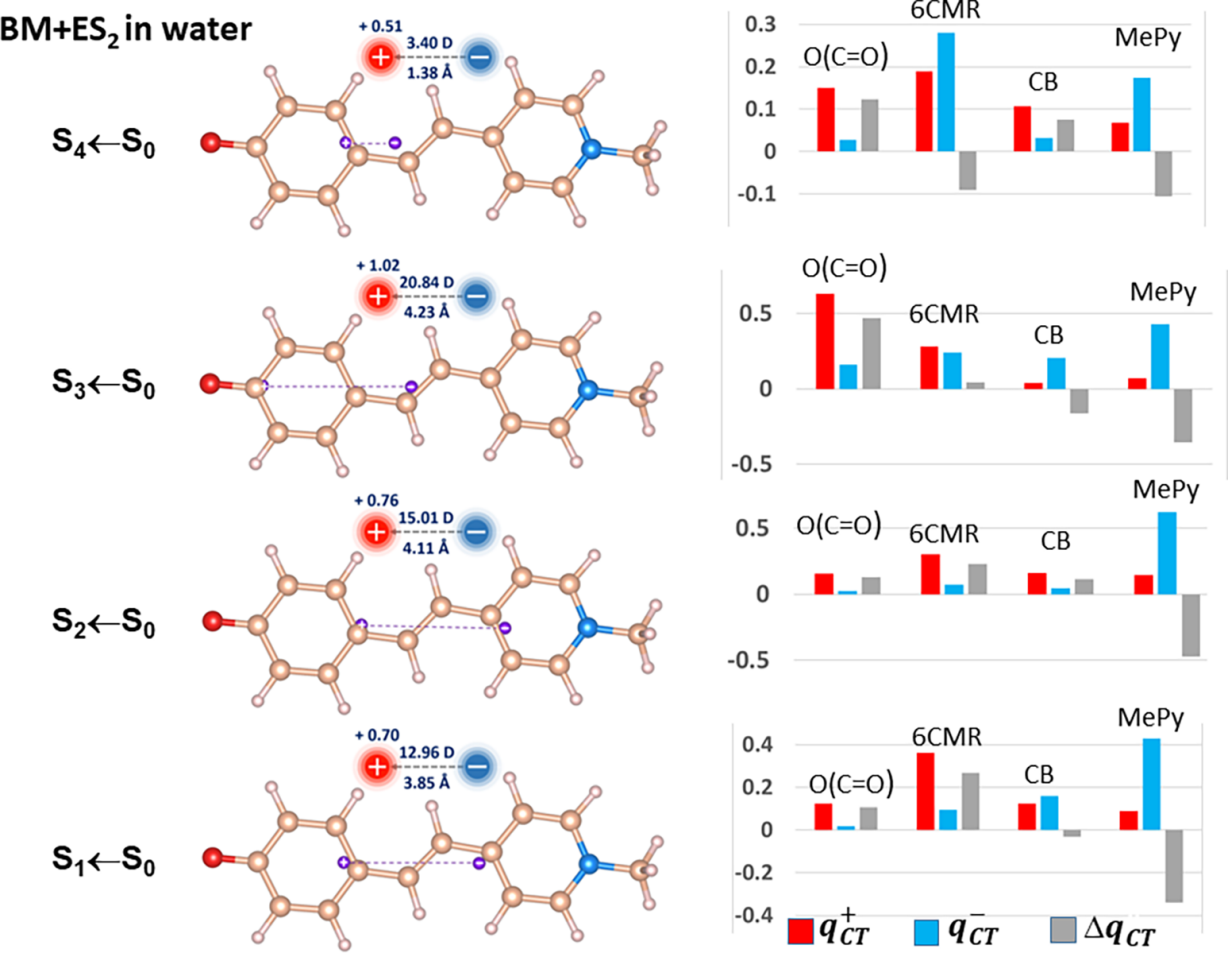
BM + ES_2_ in water: global CT indexes (left panels) and atomic groups decomposition (right panels) of the transferred charges *q*_*CT*_ upon excitation for the first S_*n*_ (*n* = 1–4) vertical excited states. The global CT indexes include the *q*_*CT*_ value (reported in e^–^ over the “+” sign in each left panel), the dipole moment change between the ground and the excited state *n,* evaluated by the DOCTRINE code and reported in Debye, D, the CT excitation length, *D*_*CT*_, given in Å, and the locations of the positive and negative centroids of the transferred charge upon excitation (the positive and negative centroids locations are indicated by purple balls in the left panels). The *q*_*CT*_ decomposition is shown both as a function of the *q*_*CT*_^+^(Ω) and of the *q*_*CT*_^–^(Ω) contributing terms. For each subdomain Ω the difference between the *q*_*CT*_^+^(Ω) and the *q*_*CT*_^–^(Ω) contributions, Δ*q*_*CT*_(Ω), is also displayed. The BM Ω subdomains considered in the atomic groups decomposition of the CT global indexes in this figure and in all following figures and tables are (i) **O (CO)**, the carbonyl oxygen atom; *(ii*) **6CMR**, the 6-Carbon Membered Ring plus its four linked H atoms; (iii) **CB**, the two C atoms of the Central Bridge plus their two linked H atoms and (iv) **MePy**, the MethylPyridine ring plus its linked 4 H atoms.

**Figure 2:**
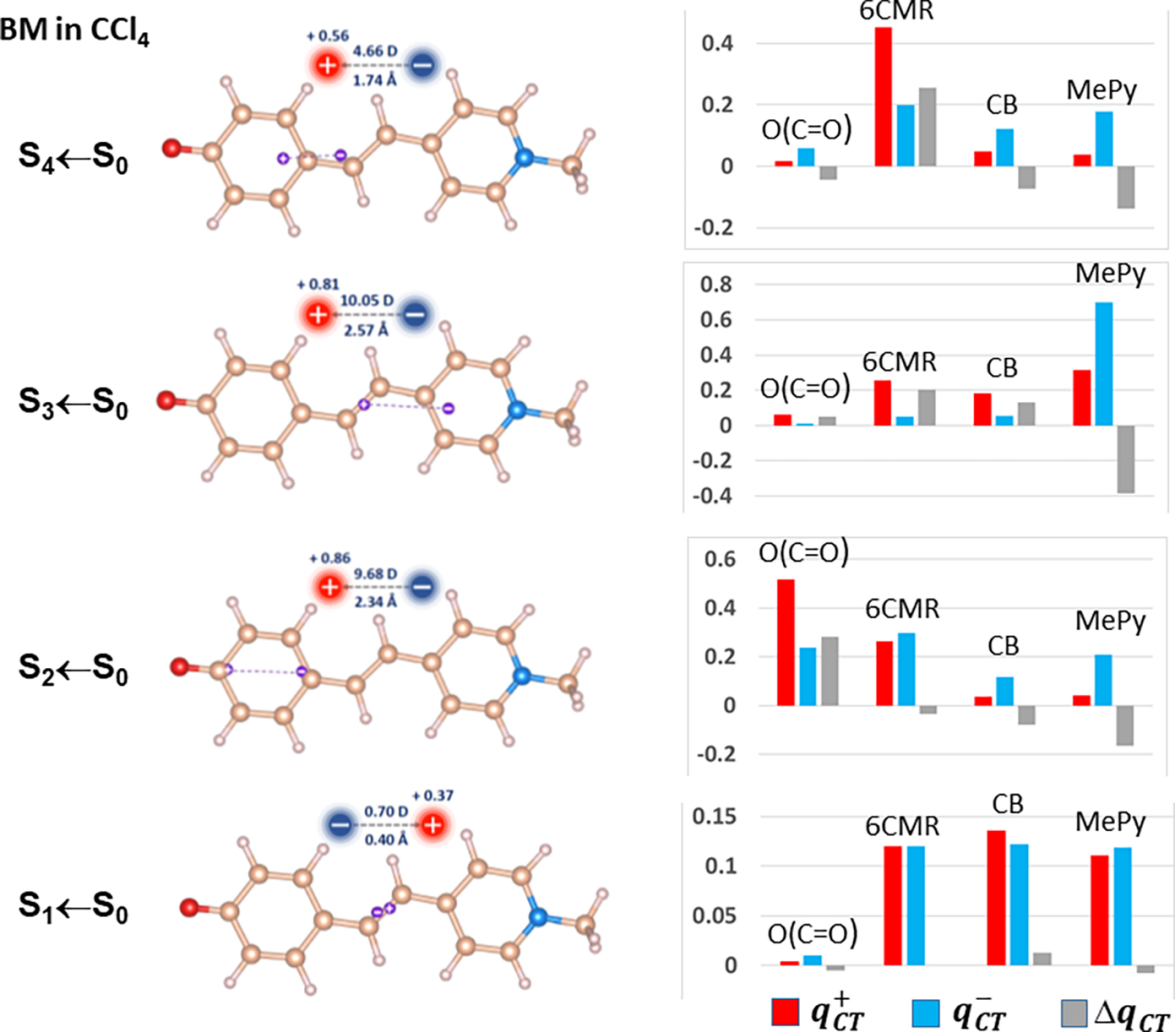
BM in CCl_4_: global CT indexes (left panels) and atomic groups decomposition (right panels) of the transferred charges *q*_*CT*_ upon excitation for the first S_*n*_ (*n* = 1–4) vertical excited states. See caption of Figure 1 for all other details.

In their left panels, Figure 1 and Figure 2 display global CT indexes relative to the first four vertical excited states S_*n*_ (*n* = 1–4) of the BM+ES_2_ in water and the BM in CCl_4_ systems, respectively. The decomposition into subdomain contributions of one of these global indexes, namely the amount of the transferred charge *q*_*CT*_ whose value is reported in e^–^ over the “+” sign in each of the left panels, is instead shown in the right panels of both figures. The *q*_*CT*_ decomposition is presented both in terms of *q*_*CT*_^+^(Ω) and *q*_*CT*_^–^(Ω) contributions. Furthermore, the difference, Δ*q*_*CT*_(Ω), between the *q*_*CT*_^+^(Ω) and the *q*_*CT*_^–^(Ω) contributions is also displayed. Table 2 gathers all these subdomain decomposition data, including, for each electronic transition, the value *A* of the sum of only positive Δ*q*_*CT*_(Ω) values,  (or, equivalently, minus the sum of the only negative Δ*q*_*CT*_(Ω) values; see eqs 9a and 9b). The other global CT indexes reported in the left panels include (i) the dipole moment change between the ground and the excited state *n,* evaluated by the DOCTRINE code and reported in Debye (D); (ii) the CT excitation length, *D*_*CT*_, given in Å, and (iii) the locations of the positive and negative centroids of the transferred charge upon excitation. The location of centroids is indicated by purple balls with a positive or negative sign identifier. As expected, in almost all excitations the electronic charge is transferred from the D to the A moiety of the BM molecule (from the left to the right of the BM molecules cases represented in the left panels of Figures 1 and 2). Nevertheless, in one single case (S_1_ ← S_0_, BM in CCl_4_) a reversed CT occurs, from the A to the D moiety of the molecule. With the exception of the S_4_ ← S_0_ excitation, the change of the dipole moment caused by the electronic transition is definitely larger in the protic water solvent (both when implicit and explicit water solvent models are adopted)^33^ than it is in the weakly polar CCl_4_ solvent. Rather than to an enhanced charge separation *q*_*CT*_, the larger ∥**μ**_*CT*_∥ is due to the much larger charge excitation length in the protic solvent, that is to the largely enhanced spatial separation of the positive and negative charge centroids (this is visible by comparing Figure 1 and 2 for the first three excited states). The excited states differ also in their specific location of the positive and negative centroid of charge. For instance, for BM + ES_2_ in water the centroids are roughly located at the extremes of the carbon bridge for the S_1_ ← S_0_ and S_2_ ← S_0_ transitions, while for the S_3_ ← S_0_ transition the positive centroid is close to the carbonyl oxygen and the negative one lies on the C atom of the bridge closer to the 6CMR. Finally, for the S_4_ ← S_0_ transition, the two centroids are at the extremes of the CC bond of the bridge closer to the 6CMR. In the case of BM in CCl_4_, the positive and negative centroids are much less separated, as previously said, yet their locations may be put in qualitative correspondence with those of BM + ES_2_ in water, provided that one takes into account that the presence of two explicit water molecules causes an energy ordering inversion, relative to the case of implicit solvent models, for the quite different (although almost energy degenerate) states 2 and 3. In other words, states 2 and 3 of BM + ES_2_ in water need to be associated and compared, respectively, to states 3 and 2 of BM in CCl_4_. The shrinking of the charge excitation length in the weakly polar solvent, relatively to BM + ES_2_ in water, is particularly evident for the S_1_ ← S_0_ transition (0.40 Å rather than 3.85 Å for BM + ES_2_ in water), which exhibits also a reversed sign, as mentioned earlier.

**Table 2:** Atomic Group Decomposition of the Transferred Charges *q*_*CT*_ upon Excitation for the First S_*n*_ (*n* = 1-4) Vertical Excited States of BM + ES_2_ in Water and of BM in CCl_4_^a^

		*q*_*CT*_^+^(Ω)				*q*_*CT*_^–^(Ω)					Δ*q*_*CT*_(Ω)			
state	*q*_*CT*_	O(CO)	6CMR	CB	MePy	O(CO)	6CMR	CB	MePy	*A*^b^	O(CO)	6CMR	CB	MePy
BM + ES_2_ in water														
S_1_ ← S_0_	0.700	0.125	**0.362**	0.124	0.089	0.019	0.095	0.158	**0.429**	0.373	0.106	0.267	–0.033	**–0.340**
S_2_ ← S_0_	0.761	0.157	**0.300**	0.160	0.145	0.025	0.070	0.046	**0.620**	0.475	0.132	0.230	0.113	**–0.475**
S_3_ ← S_0_	1.030	**0.629**	0.283	0.040	0.072	0.160	0.241	0.203	**0.427**	0.517	**0.469**	0.043	–0.163	–0.354
S_4_ ← S_0_	0.513	0.150	**0.189**	0.106	0.068	0.027	**0.280**	0.032	0.174	0.197	**0.123**	–0.091	0.075	–0.106
BM in carbon tetrachloride														
S_1_ ← S_0_	0.370	0.004	0.120	**0.136**	0.111	0.010	0.120	**0.122**	0.119	0.013	–0.005	0.000	**0.013**	–0.008
S_2_ ← S_0_	0.860	**0.518**	0.264	0.037	0.042	0.237	**0.298**	0.117	0.208	0.281	**0.281**	–0.034	–0.080	–0.166
S_3_ ← S_0_	0.814	0.061	0.255	0.184	**0.314**	0.009	0.050	0.054	**0.700**	0.387	0.052	0.204	0.130	**–0.387**
S_4_ ← S_0_	0.557	0.016	**0.453**	0.049	0.038	0.059	**0.199**	0.122	0.177	0.255	–0.043	**0.255**	–0.074	–0.138

a The *q*_*CT*_ decomposition is reported both as a function of the *q*_*CT*_^+^(Ω) and of the *q*_*CT*_^–^(Ω) contributing terms. For each subdomain Ω the difference, Δ*q*_*CT*_(Ω), between the *q*_*CT*_^+^(Ω) and the *q*_*CT*_^–^(Ω) contribution is also shown. For each excitation, the largest *q*_*CT*_^+^(Ω), *q*_*CT*_^–^(Ω) and Δ*q*_*CT*_(Ω) absolute values are highlighted in bold. See main text or the caption of Figure 1 for the labeling of the four considered molecular subdomains Ω.

b *A* =  (or, equivalently, minus the sum of the only negative Δ*q*_*CT*_(Ω) values; see eq 9a and 9b).

### Decomposition of *q*_*CT*_ into Atomic Group Subdomain Contributions (Equations 7 and 8a–8c)

Features and trends of the global CT indexes have been briefly commented above in order to analyze the further information that may be obtained from their decomposition in subdomain contributions. The decomposition of *q*_*CT*_(S_*n*_ ← S_0_) in either *q*_*CT*_^+^(Ω) or *q*_*CT*_^–^(Ω) contributions show the different roles played by the various subdomains in determining the amount of the transferred charge, according to the investigated system and electronic excitation. In the BM + ES_2_ in water, all transitions are characterized by the MePy subdomain, providing the most relevant *q*_*CT*_^–^(Ω) contribution consistently with its role of acceptor, except for S_4_ ← S_0_ excitation where the *q*_*CT*_^–^(Ω) contribution from the 6CMR prevails. On the other hand, in the sum of the *q*_*CT*_^+^(Ω) values, also yielding *q*_*CT*_, different subdomains play the major role, depending on the transition. The carbonyl oxygen dominates the *q*_*CT*_^+^(Ω) sum in the case of the excitation with the largest *q*_*CT*_, ∥**μ**_*CT*_∥, and *D*_*CT*_ magnitudes (S_3_ ← S_0_), while it is the 6CMR that plays the leading role in the other three transitions. The reconstruction of *q*_*CT*_ in terms of two alternative and different reconstructions enables us to discriminate those subdomains that concur to cause the charge separation owing to a significant increase or decrease of their electron populations, from those that marginally change their electron populations and contribute (more locally) to the charge separation because of a Δρ(***r***) polarization in their subdomain, with portions of the subdomain characterized by significantly positive Δρ(***r***) values and other portions by significantly negative Δρ(***r***) values. The former are characterized by comparatively large |Δ*q*_*CT*_(Ω)|values, while the latter have *q*_*CT*_^+^(Ω) and *q*_*CT*_^–^(Ω) contributions that tend to compensate each other, leading to comparatively small or very small |Δ*q*_*CT*_(Ω)| values. As a consequence, while MePy behaves in general as a true acceptor, hence having largely negative Δ*q*_*CT*_(Ω) values, the role of the true donor, which should have largely positive Δ*q*_*CT*_(Ω) values, is less evident and played either by the carbonyl oxygen (S_3_ ← S_0_ and S_4_ ← S_0_) or by both the 6CMR and the carbonyl oxygen but in larger measure by the former (S_1_ ← S_0_ and S_2_ ← S_0_). The CB subdomain usually seems to play a dual role in causing charge separation, polarizing itself in response to the excitation, exhibiting not negligible *q*_*CT*_^+^(Ω) and *q*_*CT*_^–^(Ω) contributions but generally small and either small positive or small negative Δ*q*_*CT*_(Ω) values. This behavior complies with the carbon bridge being a conjugation entity covalently connected to both the D and A moieties of the BM molecule and supporting the electron transport between them. The major or minor weight of the two possible situations described above may be easily appreciated by examining the ratio of *A* to *q*_*CT*_ (eq 9), that amounts to 0.53, 0.62, 0.50, and 0.38 for the first four electron transitions of BM+ES_2_ in water, respectively. The lowest value of the  ratio occurs for the S_4_ ← S_0_ transition, and the highest for S_2_ ← S_0_. In this latter transition, all subdomains are characterized by a clear dominant role (the carbonyl oxygen, the 6CMR and the CB acting as electron donors and MePy as electron acceptor), while in the former transition the MePy and in particular the 6CMR subdomains have an evident dual behavior, with part of their basins acting as electron acceptors and other not negligible portions of their basins as electron donors. Note that the trend of the  ratio does not necessarily follow that of *q*_*CT*_ and ∥**μ**_*CT*_∥ values. For instance, the largest *q*_*CT*_ and ∥**μ**_*CT*_∥ values occur for the S_3_ ← S_0_ excitation, yet the highest  value is for the S_2_ ← S_0_ transition. Indeed the location of the centroids of the transferred charge clearly indicate that the CT for the S_3_ ← S_0_ excitation is essentially taking place within the 6CMR region where, though with clamped nuclei, a switch from the benzenoid (GS) to the quinonenoid BM electron structure likely occurs (Scheme 1). Such an electronic rearrangement complies with large and similar *q*_*CT*_^+^ and *q*_*CT*_^–^ contributions for the 6CMR (0.283 and 0.241 e^–^, Table 2 and Figure 1), leading to the comparatively very small Δ*q*_*CT*_ (6CMR) value of 0.043 e^–^ and, consequently, a smaller value for the total Δ*q*_*CT*_ relative to the S_2_ ← S_0_ transition where the 6CMR behaves instead as a clear electron donor. Our reasoning is also supported by the more than doubled decrease of the C–C delocalization index (DI)^42^ of the central bond of the carbon bridge found for the S_3_ ← S_0_ relative to the S_2_ ← S_0_ transition [δDI(CB); S_3_ ← S_0_] = −0.17 vs [δDI(CB); S_2_ ← S_0_)] = −0.07. The delocalization index measures the number of electron pairs shared between two atoms, and it is close to 1 for a CC single bond and it reaches a value of almost 2 for a double CC bond. A decrease of the DI for the central CC bond of the carbon bridge implies an increase of the quinonoid relative to the benzenoid BM structure, upon excitation (see Scheme 1). As a further consequence CB behaves mostly as an electron acceptor for S_3_ ← S_0_ and more as an electron donor for the S_2_ ← S_0_ transition (Figure 1). The S_4_ ← S_0_ transition of BM+ES_2_ in water is atypical, as previously noted. Its definitely small *q*_*CT*_, ∥**μ**_*CT*_∥, and *D*_*CT*_ values are the result (Figure 1 and Table 2) of an alternate D–A–D–A (D = donor; A = acceptor) D/A behavior of the subdomains (from left to right in Figure 1) whereas a more neat spatial separation of the D and A behavior in the molecule is found to occur for S_3_ ← S_0_ and S_1_ ← S_0_ (D–D–A–A) or for S_2_ ← S_0_ (D–D–D–A) excitations.

Let us examine now the S_*n*_ (*n* = 1–4) vertical excited states of BM in CCl_4_ in comparison to those already discussed for the water solvent case. The  ratio (from data in Table 2), for the first four electron transitions of BM in CCl_4_ amounts to just 0.04, 0.33, 0.48, and 0.46, so it is lower or much lower than for BM + ES_2_ in water, except for the S_4_ ← S_0_ transition that is quite anomalous for this latter solvent model. The generally lower  ratio, as well as the much lower ∥**μ**_*CT*_∥ and *D*_*CT*_ values for corresponding states in the BM in CCl_4_ vs BM + ES_2_ in water are but the consequence of the general D and A dual behavior of subdomains in the former system. The case of the S_1_ ← S_0_ excitation (Figure 2 and Table 2) is particularly revealing with all subdomains having negligible Δ*q*_*CT*_(Ω) values. This is the only case, among the eight here investigated, where the MePy subdomain has a totally marginal role as a donor (Δ*q*_*CT*_ = −0.008 e^–^) notwithstanding the significant, yet almost equal in value, *q*_*CT*_^+^ and *q*_*CT*_^–^ contributions (0.111 and 0.119 e^–^, respectively). The negligible Δ*q*_*CT*_(MePy) value, along with the carbonyl oxygen playing the prevalent role of weak acceptor rather than strong donor (Δ*q*_*CT*_(O(CO) = −0.005, Table 2) is responsible of the, albeit limited, CT inversion. The transitions with the larger ∥**μ**_*CT*_∥ and *D*_*CT*_ values have instead well separate donor and acceptor regions, with D–A–A–A (S_2_ ← S_0_) or D–D–D–A (S_3_ ← S_0_) D/A patterns (Figure 2, left panels, from left to right) for the selected subdomains. The (S_4_ ← S_0_) transition has the lowest ∥**μ**_*CT*_∥ and *D*_*CT*_ values among the excitations with direct CT and, not unexpectedly, has an almost alternate D/A pattern, namely A–D–A–A, with (O(CO) playing the role of the acceptor rather than donor. This change from the usual role for the O(CO) corresponds to an enhanced weight of the benzenoid rather than of the quinonic BM structure upon excitation. This is corroborated by a 0.03 e^–^ increase rather than a decrease of the electron population of the oxygen atom and a small 0.01 increase, rather than a clear decrease, of the DI value of the carbon bridge central CC bond in the excited state.

### Decomposition of ∥**μ**_*CT*_∥ into Atomic Group Subdomain Contributions (According to Equations 16a and 16b)

**Figure 3:**
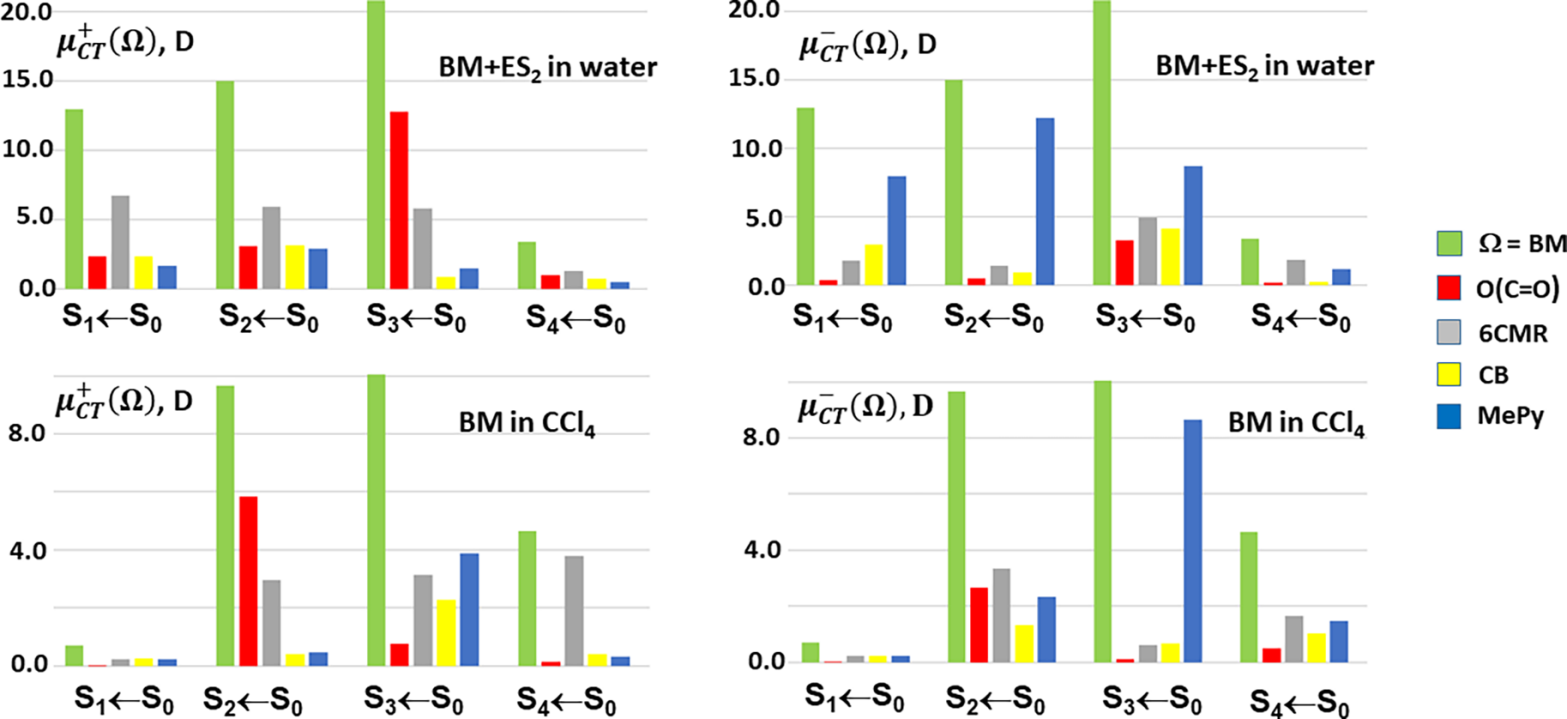
Atomic groups decomposition of the norm of the dipole moment change, ∥**μ**_*CT*_∥ (S_*n*_ ← S_0_), between the ground and the excited state *n*, according to eqs 16a and 16b. The total dipole moment change **μ**_*CT*_^+^(Ω = all atoms of the BM molecule) for each excitation is represented by the pale green bar. Both the **μ**_*CT*_^+^(Ω) and the **μ**_*CT*_^–^(Ω) contributions to the total dipole moment change are shown, where Ω is any of the four subdomains of the BM molecule that have been considered in the analysis (see text). Note that a few contributions are small and hardly detectable in the figure. Their numerical values are listed in Table 3.

Figure 3 and Table 3 display and report the decomposition in subdomains contributions of the norm of the dipole moment change, ∥**μ**_*CT*_∥ (S_*n*_ ← S_0_), between the ground and the excited state *n*. In the Figure and Table 3, the ∥**μ**_*CT*_∥ decomposition is performed according to eqs 16a and 16b; i.e., only the dependence of ∥**μ**_*CT*_∥ on *q*_*CT*_ is taken into account. The total dipole moment change for each excitation is represented by a pale green bar and both the **μ**_*CT*_^+^(Ω) and the **μ**_*CT*_^–^(Ω) contributions to the total dipole moment change are shown for all four subdomains Ω considered in our analysis.

**Table 3:** Atomic Group Decomposition of the Norm of the Dipole Moment Change, ∥**μ**_*CT*_∥ (S_*n*_ ← S_0_) (Given in Debye, D), for the First S_*n*_ (*n* = 1-4) Vertical Excited States of BM + ES_2_ in Water and of BM in CCl_4_^a^

		**μ**_*CT*_^+^(Ω), D				**μ**_*CT*_^–^(Ω), D			
state	∥**μ**_*CT*_∥, D	O(CO)	6CMR	CB	MePy	O(CO)	6CMR	CB	MePy
BM + ES_2_ in water									
S_1_ ← S_0_	12.958	2.308	**6.700**	2.301	1.648	0.346	1.762	2.919	**7.938**
S_2_ ← S_0_	15.013	**3.093**	**5.912**	3.150	2.858	0.496	1.379	0.912	**12.237**
S_3_ ← S_0_	20.835	**12.786**	5.763	0.818	1.469	3.246	4.896	4.128	**8.675**
S_4_ ← S_0_	3.399	0.992	**1.253**	0.704	0.449	0.180	**1.856**	0.210	1.150
BM in carbon tetrachloride									
S_1_ ← S_0_	0.704	0.008	0.229	**0.258**	0.211	0.018	0.227	**0.233**	0.226
S_2_ ← S_0_	9.676	**5.827**	2.967	0.415	0.467	2.667	**3.353**	1.318	2.337
S_3_ ← S_0_	10.049	0.755	3.144	2.277	**3.874**	0.107	0.623	0.672	**8.646**
S_4_ ← S_0_	4.663	0.134	**3.799**	0.407	0.323	0.496	**1.663**	1.024	1.480

a Both the **μ**_*CT*_^+^(Ω) and the **μ**_*CT*_^–^(Ω) contribution to ∥**μ**_*CT*_∥ are listed in the table. They are calculated according to eqs 16a and 16b that takes into account only the ∥**μ**_*CT*_∥ dependence on *q*_*CT*_. For each excitation, the largest **μ**_*CT*_^+^(Ω) and the **μ**_*CT*_^–^(Ω) values are highlighted in bold.

In the first three excited states of BM + ES_2_ in water, the ∥**μ**_*CT*_∥ reconstruction in terms of **μ**_*CT*_^+^(Ω) is dictated by the carbonyl oxygen and by the 6CMR contributions (representing 70, 60, and 89% of the total dipole, respectively). The 6CMR contribution is the largest one for the first two excited states, whereas it is the carbonyl oxygen that dominates the third excitation with a 61% contribution to what represents the largest ∥**μ**_*CT*_∥ value (20.8 D) among the eight investigated cases. Not unexpectedly, the decrease of the carbonyl oxygen electron population along the series of the first three excited states (from 9.29 in the GS to 9.18, 9.16, and eventually 8.78 e^–^ in the third excited state) parallels the increase (from 18 to 21 to 61%) of its **μ**_*CT*_^+^(Ω) contribution to ∥**μ**_*CT*_∥. The fourth excited state of BM+ES_2_ in water has a small ∥**μ**_*CT*_∥ value (3.4 D) with comparable **μ**_*CT*_^+^(Ω) contributions from the four subdomains. The ∥**μ**_*CT*_∥ reconstructions in terms of **μ**_*CT*_^–^(Ω) contributions differ from those using the **μ**_*CT*_^+^(Ω) ones, as expected. The first two excited states are governed by the MePy contribution amounting to 61 and 82% of the total, while in the third state MePy exhibits still the largest contribution (42%), but also the other 3 subdomains give substantial and similar to each other contributions summing up to the remaining 58%. Therefore, for the excitation with the largest ∥**μ**_*CT*_∥ value, the **μ**_*CT*_^+^(Ω) reconstruction is evidently governed by the carbonyl oxygen, whereas all four subdomains concur with relevant weights to the ∥**μ**_*CT*_∥ reconstruction in terms of **μ**_*CT*_^–^(Ω), as clearly anticipated by the associated *q*_*CT*_ decomposition using *q*_*CT*_^–^(Ω). The ∥**μ**_*CT*_∥ reconstructions for BM in CCl_4_ look different from those of the BM+ES_2_ in water. Yet, those for states 2 and 3 are qualitatively similar if the mentioned interchange between states 2 and 3 is taken into account. The first excited state exhibits similar and small contributions from all subdomains in both the **μ**_*CT*_^+^(Ω) and **μ**_*CT*_^–^(Ω) reconstructions except the carbonyl oxygen subdomain that gives an almost zero contribution in both sums. For the fourth excited state, the 6CMR dominates the ∥**μ**_*CT*_∥ reconstruction in terms of **μ**_*CT*_^+^(Ω), while in that based on **μ**_*CT*_^–^(Ω) the 6CMR still provides the largest contribution, but both the CB and the MePy moieties are also contributing in a significant way.

### Decomposition of the CT Excitation Length into Atomic Group Subdomain Contributions (Equations 10–12)

**Figure 4:**
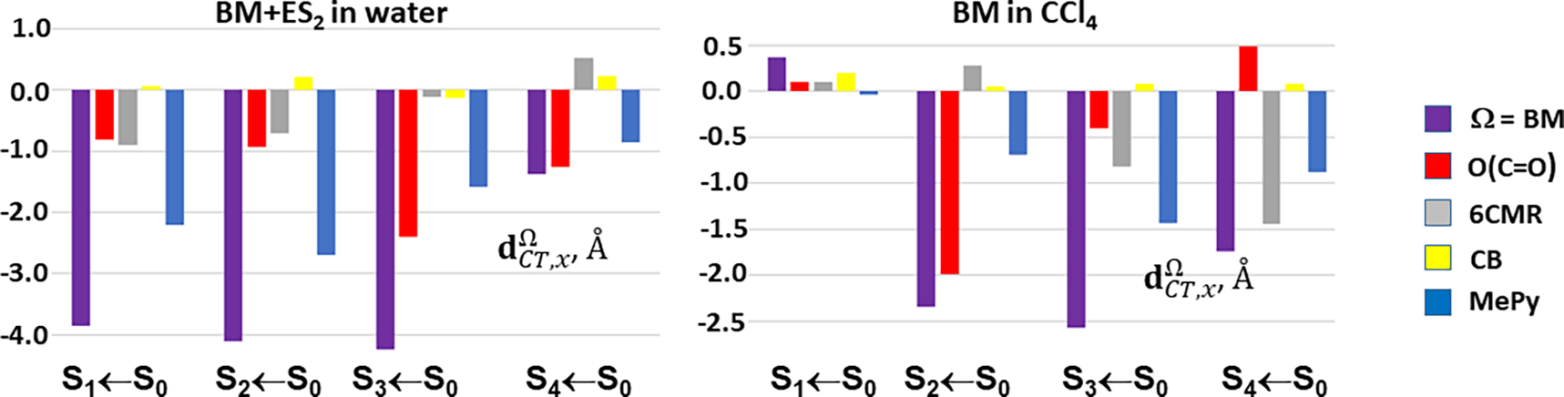
Atomic groups decomposition of the *x* component, **d**_*CT*, *x*_^Ω^ (given in Å), of the excitation length vector, ***D***_*CT*_, for the first S_*n*_ (*n* = 1–4) vertical excited states of BM + ES_2_ in water and of BM in CCl_4_. The *x* axis is almost collinear and, for all cases but one, antiparallel to ***D***_*CT*_.

Figure 4 and Table 4, respectively display and report the atomic groups decomposition of the *x* component, **d**_*CT*, *x*_^Ω^, of the excitation length vector, ***D***_*CT*_, for the first S_*n*_ (*n* = 1–4) vertical excited states of BM + ES_2_ in water and of BM in CCl_4_. Only the *x* component, **d**_*CT*, *x*_^Ω^, of the atomic groups contribution to the excitation length vector ***D***_*CT*_ is shown in the Figure 4 and listed in Table 4 since the *x* axis is almost collinear and, for all cases but one (S_1_ ← S_0_ of BM in CCl_4_), antiparallel to ***D***_*CT*_. The degree of collinearity is measured by *α*_*x*_ (see eq 13), i.e., by the *x*-axis direction cosine of ***D***_*CT*_**=** (**R**^+^ – **R**^–^), which is listed in the Table 4 for each excitation. The *x* axis is taken as directed from the left to the right of the molecule whereas **R**^+^ – **R**^–^ is almost oppositely directed if the locations of the centroids of positive and negative transferred charge reflect a photoinduced CT from the O(CO) and the 6CMR to the MePy moiety of the BM molecule. Apart S_1_ ← S_0_ of BM in CCl_4_, this is indeed the common situation in the eight reported cases, and therefore, the *x* component, **d**_*CT*, *x*_^Ω^, of the excitation length vector, ***D***_*CT*_, is generally negative (Figure 4 and Table 4). The corresponding subdomain contributions **d**_*CT*, *x*_^Ω^ are also typically negative, but in a number of cases, especially for BM in CCl_4_, they may be positive for some subdomains, that hence tend to contrast the global charge transfer excitation length. More generally, a **d**_*CT*, *x*_^Ω^sign opposite to the ***D***_*CT*_ sign signals that the subdomain Ω opposes the global charge transfer excitation length and acts so as to diminish it. The ***g***_*CT*, *x*_^Ω^ values, measuring the relative weight of a **d**_*CT*, *x*_^Ω^ contribution and given by the ratio of **d**_*CT*, *x*_^Ω^ to the CT excitation length *D*_*CT*_ (see eq 12), are also reported in Table 4 and have clearly the same sign as **d**_*CT*, *x*_^Ω^. When also ***g***_*CT*, *x*_^Ω^ is almost collinear to ***D***_*CT*_ (which is always the case in our investigated cases) the ***g***_*CT*, *x*_^Ω^ value multiplied by 100 expresses the percentage contribution of the subdomain to the observed excitation length. In the first two transitions of BM + ES_2_ in water, it is the MePy subdomain that governs *D*_*CT*_ (***g***_*CT*, *x*_^Ω^, with Ω = MePy being −0.57 and −0.66, respectively) while it is the carbonyl oxygen that takes this role for the two subsequent excitations (***g***_*CT*, *x*_^Ω^ with Ω = O(CO) being −0.57 and −0.92). With the exception of S_4_ ← S_0_ the 6CMR appreciably concurs to increase the excitation length, but in the case of the S_4_ ← S_0_ it clearly contrasts it. Not unexpectedly, this behavior is associated with the 6CMR behaving more as an A rather than a D for this excitation (Figure 1). The contributions from the CB are generally marginal (***g***_*CT*, *x*_^Ω^ being equal to 0.02, 0.05, −0.03, and 0.16 for the first four excited states and for Ω = CB) and, in but one case, all contrasting the global excitation length. In the case of BM in CCl_4_, a different subdomain plays the major role for each excitation, namely CB for the first (***g***_*CT*, *x*_^Ω^ = 0.50),O(CO) for the second one (***g***_*CT*, *x*_^Ω^ = −0.89), MePy for the third transition (***g***_*CT*, *x*_^Ω^ = −0.56), and finally, the 6CMR for the fourth one (***g***_*CT*, *x*_^Ω^ = −0.83).

**Table 4:** Atomic Group Decomposition of the CT Excitation Length, *D*_*CT*_, Given in Å, for the First S_*n*_ (*n* = 1-4) Vertical Excited States of BM + ES_2_ in Water and of BM in CCl_4_^a^

				**d**_*CT*, *x*_^Ω^, Å				***g***_*CT*, *x*_^Ω^, Å^c^			
state	*D*_*CT*_, Å	***D***_*CT*, *x*_, Å	*α*_*x*_^b^	O(CO)	6CMR	CB	MePy	O(CO)	6CMR	CB	MePy
BM + ES_2_ in water											
S_1_ ← S_0_	3.855	–3.855	–1.000	–0.805	–0.897	0.058	**-2.210**	–0.209	–0.233	0.015	**-0.574**
S_2_ ← S_0_	4.108	–4.108	–1.000	–0.926	–0.702	0.215	**-2.696**	–0.226	–0.171	0.052	**-0.656**
S_3_ ← S_0_	4.236	–4.236	–1.000	**-2.406**	–0.112	–0.134	–1.584	**-0.568**	–0.026	–0.032	–0.374
S_4_ ← S_0_	1.378	–1.378	–1.000	**-1.264**	0.525	0.222	–0.862	**-0.917**	0.387	0.161	–0.625
BM in carbon tetrachloride											
S_1_ ← S_0_	0.396	0.373	0.941	0.101	0.107	**0.198**	–0.033	0.254	0.271	**0.501**	–0.085
S_2_ ← S_0_	2.342	–2.341	–1.000	**-1.982**	0.282	0.051	–0.692	**-0.846**	0.120	0.022	–0.295
S_3_ ← S_0_	2.571	–2.568	–0.999	–0.403	–0.813	0.079	-**1.431**	–0.157	–0.316	0.031	**-0.557**
S_4_ ← S_0_	1.744	–1.741	–0.998	0.487	**-1.439**	0.085	–0.874	0.279	**-0.825**	0.049	–0.501

a Only the *x* component, **d**_*CT*, *x*_^Ω^, of the atomic groups contribution to the excitation length vector ***D***_*CT*_ is listed since the *x* axis is almost collinear and, for all cases but one, anti-parallel to ***D***_*CT*_. Additionally, the ***g***_*CT*, *x*_^Ω^ values given by the ratio of **d**_*CT*, *x*_^Ω^ to the CT excitation length *D*_*CT*_, are listed in the table. For each excitation, the largest **d**_*CT*, *x*_^Ω^ and ***g***_*CT*, *x*_^Ω^ absolute values are highlighted in bold.

b *α*_*x*_ is the *x*-axis direction cosine of ***D***_*CT*_**=** (**R**^+^ – **R**^–^) (see eq 13)

c ***g***_*CT*, *x*_^Ω^ is a dimensionless quantity providing a measure of the **d**_*CT*, *x*_^Ω^ length relative to the *D*_*CT*_ length (see eq 12). A negative sign of ***g***_*CT*, *x*_^Ω^ means that **d**_*CT*, *x*_^Ω^ is oppositely directed to (**R**^+^ – **R**^–^)_*x*_

### Decomposition of the **μ**_*CT*_ Vector into Its Intra- and Intersubdomain Contributions (Equation 15)

**Figure 5:**
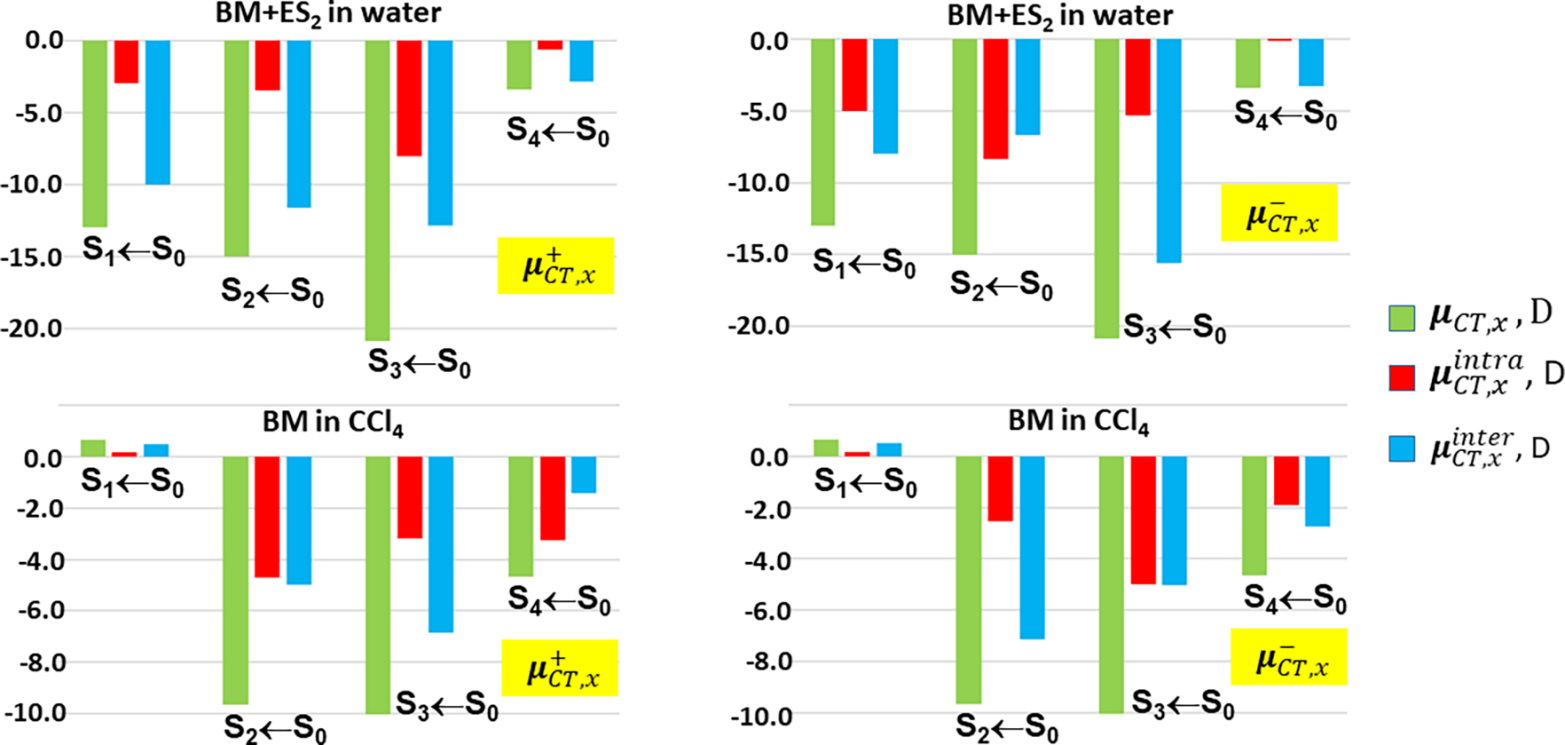
Decomposition of the *x*-component (in Debye, D) of the **μ**_*CT*_ vector into an *intra*-subdomains contribution, μ_*CT*_^*intra*^, and an *inter*-subdomains contribution, μ_*CT*_^*inter*^, for the first S_*n*_ (*n* = 1–4) vertical excited states of BM+ES_2_ in water and of BM in CCl_4_. The other two **μ**_*CT*_ vector components (*y*, *z*) have, comparatively, negligible values. The subdomains contributions are listed for both the **μ**_*CT*, *x*_^+^ and **μ**_*CT*, *x*_^–^ vector component subdomain decompositions.

Figure 5 and Table 5 respectively show and detail the decomposition of the *x*-component of the **μ**_*CT*_ vector into its *intra*-subdomains contribution, **μ**_*CT*_^*intra*^, and its *inter*-subdomains contribution **μ**_*CT*_^*inter*^ for the first S_*n*_ (*n* = 1–4) vertical excited states of BM+ES_2_ in water and of BM in CCl_4_. The decomposition of the **μ**_*CT*_ vector is afforded through eq 15 that, differently from eqs 16a and 16b and from what was reported in Figure 3 and Table 3, takes into account the dependence of ∥**μ**_*CT*_∥ on both *q*_*CT*_ and *D*_*CT*_ and not just *q*_*CT*_ The other two **μ**_*CT*_ vector components (*y,z*) have comparatively negligible values due to the fact that the excitation length vector ***D***_*CT*_ is almost parallel or antiparallel to the *x* axis in all excitations (see Table 4) and are thus not reported. The *intra and the inter* subdomains contributions are listed for both matrices *M*^*x*,+^ and *M*^*x*,–^ (eq 15), i.e. for both **μ**_*CT*, *x*_^+^ and **μ**_*CT*, *x*_^–^ vector component subdomain decompositions. In the following, we will therefore refer to both **μ**_*CT*, *x*_^*intra*,+^, **μ**_*CT*, *x*_^*inter*,+^, and **μ**_*CT*, *x*_^*intra*,–^, **μ**_*CT*, *x*_^*inter*,–^ contributions, respectively. In the case of BM + ES_2_ in water the **μ**_*CT*_^*inter*^ contribution dominates the *x*-component of the **μ**_*CT*_vector, being in all cases but one from 62 to 96% of the total value, according to the examined excited state and according to whether **μ**_*CT*, *x*_^+^ and **μ**_*CT*, *x*_^–^ vector components are considered. Only for S_2_ ← S_0_, **μ**_*CT*, *x*_^*intra*,–^ is larger in magnitude than **μ**_*CT*, *x*_^*inter*,–^ but **μ**_*CT*, *x*_^*inter*,+^ is much larger than **μ**_*CT*, *x*_^*intra*,+^. So, it looks like, in general, the larger contributions to the dipole moment change upon excitation come from coupled terms where the transferred charge contribution of a subdomain is coupled with the excitation charge transfer length contribution of a different subdomain. Accordingly, local contributions to the dipole moment change are less effective than those that are delocalized over two subdomains. The situation for BM in CCl_4_ is partly different. The **μ**_*CT*_^*inter*^ and **μ**_*CT*_^*intra*^ contributions are more similarly relevant, their *x*-component of the **μ**_*CT*_ vector ranging between 30/75% and 25/70% of the total value, respectively.

**Table 5:** Decomposition of the *x*-Component (in Debye, D) of the **μ**_*CT*_ Vector into an *Intra*subdomains Contribution, μ_*CT*_^*intra*^, and an *Inter*subdomains Contribution μ_*CT*_^*inter*^, According to eq 15, for the First S_*n*_ (*n* = 1–4) Vertical Excited States of BM + ES_2_ in Water and of BM in CCl_4_^a^

state	**μ**_*CT*, *x*_, D	**μ**_*CT*, *x*_^*intra*,+^, D^b^	**μ**_*CT*, *x*_^*inter*,+^, D^b^	**μ**_*CT*, *x*_^*intra*,–^, D^b^	**μ**_*CT*, *x*_^*inter*,–^, D^b^
BM + ES_*2*_ in water					
S_1_ ← S_0_	–12.963	–2.953 (−22.7)	–10.010 (−77.3)	–4.992 (−38.5)	–7.979 (−61.5)
S_2_ ← S_0_	–15.017	–3.418 (−22.8)	–11.599 (−77.2)	–8.331 (−55.5)	–6.697 (−44.5)
S_3_ ← S_0_	–20.857	–7.997 (−38.4)	–12.854 (−61.6)	–5.351 (−25.7)	–15.610 (−74.3)
S_4_ ← S_0_	–3.399	–0.600 (−17.7)	–2.799 (−82.3)	–0.143 (−4.2)	–3.253 (−95.8)
BM in carbon tetrachloride					
S_1_ ← S_0_	0.663	0.175 (26.4)	0.488 (73.6)	0.164 (24.7)	0.500 (75.4)
S_2_ ← S_0_	–9.674	–4.703 (−48.6)	–4.971 (−51.4)	–2.516 (−26.0)	–7.157 (−74.0)
S_3_ ← S_0_	–10.037	–3.198 (−31.8)	–6.838 (−68.1)	–5.005 (−49.8)	–5.030 (−50.1)
S_4_ ← S_0_	–4.655	–3.240 (−69.5)	–1.415 (−30.4)	–1.925 (−41.3)	–2.730 (−58.6)

a The other two **μ**_*CT*_ vector components (*y*, *z*) are not reported as are, in comparison, negligible in value. The *intra and the inter* subdomains contributions are listed for both matrices *M*^*x*,+^ and *M*^*x*,–^ (see text), *i.e.* for both **μ**_*CT*, *x*_^+^ and **μ**_*CT*, *x*_^–^ vector components subdomain decompositions.

b In parentheses, the percentage values relative to the associated **μ**_*CT*, *x*_value are reported.

### The Subdomain Dipole Moment Matrices (Equation 15)

The decomposition, discussed above, of the **μ**_CT_ vector into its intra- and intersubdomains contributions is a *condensed form* representation of the complete information on the **μ**_CT_ vector contained in the *M*^*i*,+^ and *M*^*i*,–^ matrices (eq 15). Table 6 lists the values of the elements of the matrices *M*^*x*,+^ and *M*^*x*,–^ (eq 15) relative to the S_4_ ← S_0_ transition in the systems BM + ES_2_ in water and BM in CCl_4_. The corresponding matrices for the S_*n*_ ← S_0_ (*n* = 1–3) transitions are reported in the Supporting Information, Tables S1–S3. As the excitation length vector ***D***_*CT*_ is almost parallel or antiparallel to the *x* axis in all excitations, the elements of the matrices of the components *y* and *z* of the **μ**_*CT*_ vector have values that are, in general, comparatively negligible in magnitude relative to those of the *x* component matrix and are thus not reported. The sum of the elements on the diagonal of the matrix *M*^*x*,+^ (or *M*^*x*,–^) (those with pink background) corresponds to the *intra*-subdomains contribution, μ_*CT*_^*intra*^, to **μ**_*CT*, *x*_^+^ (or to **μ**_*CT*, *x*_^–^) while the sum of the out of diagonal elements (those with light blue background) corresponds to the *inter*-subdomains contribution, μ_*CT*_^*inter*^, to **μ**_*CT*, *x*_^+^ (or to **μ**_*CT*, *x*_^–^) (eq 15). Analysis of the values of matrices *M*^*x*,+^ and *M*^*x*,–^ enables us to reveal which subdomains are more effective in contributing to the μ_*CT*_^*intra*^ term and which are more strongly coupled together and thus largely contributing to the μ_*CT*_^*inter*^ term. The sign of the matrices elements is also clearly relevant, since a sign opposite to that of the dipole moment indicates that the associated contribution is opposing rather than concurring to the observed **μ**_*CT*_.

**Table 6:**
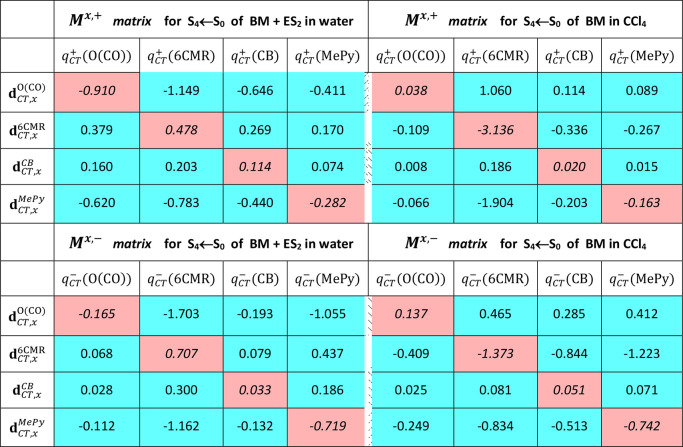
Values (in Debye, D) of the Elements of the Matrices *M*^*x*,+^ and *M*^*x*,–^ Relative to the S_4_ ← S_0_ Transition in the Systems: BM + ES_2_ in Water and BM in CCl_4_^a^

a The corresponding matrices for the S_*n*_ ← S_0_ (*n* = 1-3) transitions are reported in the Supporting Information, Tables S1–S3. The elements of the matrices of the components *y* and *z* of the **μ**_*CT*_ vector have values that are, in general, comparatively much smaller in magnitude than those of the *x* component matrix. The sum of the elements on the diagonal of the matrix *M*^*x*,+^ (or *M*^*x*,–^) (namely, those with pink background) corresponds to the *intra*subdomain contribution, μ_*CT*_^*intra*^, to **μ**_*CT*, *x*_^+^ (or to **μ**_*CT*, *x*_^–^) while the sum of the out of diagonal elements (those with light blue background) corresponds to the *inter*subdomain contribution, μ_*CT*_^*inter*^, to **μ**_*CT*, *x*_^+^ (or to **μ**_*CT*, *x*_^–^) (eq 15).

Considering the elements of the *M*^*x*,+^ and *M*^*x*,–^ matrices relative to the S_4_ ← S_0_ transition in BM+ES_2_ in water, it is easy to observe that the dominant magnitudes of the **μ**_*CT*, *x*_^*inter*,+^ and **μ**_*CT*, *x*_^*inter*,–^ vectors (82.3 and 95.8%, respectively, Table 5) are due to the mixed inter subdomain terms of the first row, involving **d**_*CT*, *x*_^O(CO)^ and, among *q*_*CT*_^+^(Ω), in particular *q*_*CT*_^+^(6CMR) and those of the fourth row, involving **d**_*CT*, *x*_^*MePy*^ and, among *q*_*CT*_^+^(Ω), again *q*_*CT*_^+^(6CMR), in particular. Other mixed terms in the second and third row are found to oppose the observed **μ**_*CT*_ and their total sum amounts to 1.25 and 1.10 D for *M*^*x*,+^ and *M*^*x*,–^ relative to **μ**_*CT*, *x*_^*inter*,+^ and **μ**_*CT*, *x*_^*inter*,–^ magnitudes of −2.8 and −3.3 D (Table 5). In overall, it is the transferred charge originating from the 6CMR and the CT excitation length due to the carbonyl oxygen and to the MePy subdomain that mostly contributes to the dipole moment change for this electron transition. Nonetheless, other mixed terms are also found to be relevant, some of them favoring and some others opposing the dipole moment change.

In the case of the S_4_ ← S_0_ transition of BM in CCl_4_, the magnitudes of the **μ**_*CT*, *x*_^*intra*,+^, **μ**_*CT*, *x*_^*inter*,+^, **μ**_*CT*, *x*_^*intra*,–^, **μ**_*CT*, *x*_^*inter*,–^ vectors are comparable to each other (69.5, 30.4, 41.3, and 58.6%, respectively), so both the diagonal and the out of diagonal elements of matrices *M*^*x*,+^ and *M*^*x*,–^ play a relevant role in determining the dipole moment change upon electron transition. Concerning *M*^*x*,+^, are the intrasubdomain **d**_*CT*, *x*_^6CMR^·*q*_*CT*_^+^(6CMR) and the intersubdomain **d**_*CT*, *x*_^*MePY*^·*q*_*CT*_^+^(6CMR) contributions that mostly favor the dipole moment change (−3.136 and −1.904 D, respectively, to be compared to the total **μ**_*CT*, *x*_magnitude of −4.655 D, Table 5), while it is the mixed term **d**_*CT*, *x*_^O(CO)^·*q*_*CT*_^+^(6CMR), with a value of 1.060 D, that mostly opposes the dipole moment change. Concerning *M*^*x*,–^, the terms of the second row involving **d**_*CT*, *x*_^6CMR^ and *q*_*CT*_^+^(Ω = 6CMR, CB, and MePy) and those of the fourth row involving **d**_*CT*, *x*_^*MePY*^ and *q*_*CT*_^+^(Ω = 6CMR, CB, and MePy) play the major role in favoring the observed dipole moment change. Other contributions are less relevant in favoring the dipole or act to oppose it to various extents, the most effective, with a value of 1.060 D, being **d**_*CT*, *x*_^O(CO)^·*q*_*CT*_^+^(6CMR).

The *M*^*x*,+^ and *M*^*x*,–^ matrices listed in Table 6 serve just as an example of the chemical insight these matrices can provide on the origin of the observed dipole moment change. The other examples reported in Tables S1–S3, for the remaining six investigated electron transitions, have a similar purpose and may be analyzed following the same lines sketched above for the only cases of the S_4_ ← S_0_ transition in the systems BM+ES_2_ in water and BM in CCl_4_. For the sake of space, their discussion is therefore not reported. Just note that the transition with the largest dipole moment change (S_3_ ← S_0_ in BM + ES_2_ in water, Table S3) has all the *M*^*x*,+^ and *M*^*x*,–^ elements bearing the same sign, thereby indicating that all intra- and intersubdomain contributions jointly cooperate to yield the observed dipole moment change.

## Conclusions

We have presented a new method for decomposing the Le Bahers, Adamo, Ciofini (LBAC) Charge Transfer Excitations global indexes^9^ into molecular subdomains contributions, and a software package for the application of the method has been coded. Analogously to the LBAC original model, the subdomain decomposition of the CT indexes is made in the real space, using the rearrangement electron density given by the difference between the electron distribution in the excited state and that in the ground state. Working in real space has the special advantage that the *intra*- and *inter*fragments contributions to the CT indexes, analogous to their global sums, are much less basis set and method dependent, provided that both the basis set and the quantum mechanical adopted method are of a sufficient quality.

Our method applies to any fuzzy or to any disjointed exhaustive partitioning of the real space. However, using a definition of chemically relevant molecular subdomains based on the Atoms in Molecules Bader basins has the important advantage of associating *intra*- or *inter*subdomain contributions to rigorously defined quantum objects, yet bearing a clear chemical meaning.

The developed method allows for a quantitative evaluation of the subdomain contributions to the charge transfer, the charge transfer excitation length, and the dipole moment change upon excitation. All these global indexes may be obtained either from the electron density increment or the electron density depletion upon excitation. However, the subdomain contributions obtained from the two distributions generally differ, therefore allowing one to distinguish whether the contribution to a given property of a given subdomain is dominated by one of the two distributions or if both are playing an important role.

As a *toy* system for the first application of our model, a typical compound [D−π–A, π = conjugated bridge], belonging to the merocyanine dyes family has been selected and the first four excited states of BM in a strongly polar protic solvent and in a weakly polar solvent have been scrutinized. It is remarkable to note how the subdomain contributions of the transferred charge and of the CT excitation length are able to reveal the causes behind the distinct features of the investigated excitations. The global CT indexes have often underlying chemical motifs that would be hardly possible to quantify or to even imagine without decomposing them in suitable concurring or opposing contributions. Since the subdomain contributions of the transferred charge and of the CT excitation length jointly concur also to determine the change in the dipole moment upon electron transition, the subdomain contributions patterns represent a precious and chemically insightful representation of the photoexcited transitions.

In overall, we hope that our method may provide another useful and distinct tool among the many that have been already developed to help practitioners in the field of light-driven charge transfer processes and that the subdomain decomposition of the CT indexes may serve to further deepen our detailed understanding of these processes.

## Supporting Information Available

Tables S1–S3, values (in Debye, D) of the elements of the matrices *M*^*x*,+^ and *M*^*x*,–^ relative to the S_*n*_ ← S_0_ (*n* = 1–3) transitions in the systems BM + ES_2_ in water and BM in CCl_4_ (PDF)
