# Supplementary material for: Atomic Group Decomposition of Charge Transfer Excitation Global Indexes
Source: J Phys Chem A. 2022 Sep 2;126(36):6314–28. doi: 10.1021/acs.jpca.2c04607 (PMC9483980; doi:10.1021/acs.jpca.2c04607)
Supplement: Supplementary file 1 — jp2c04607_si_001.pdf [file jp2c04607_si_001.pdf]

Supporting Information for Publication

Atomic Groups Decomposition of Charge Transfer Excitations Global Indexes

Carlo Gatti *[a,b], Yann Danten [c] and Christine Frayret [d]

a) CNR SCITEC, CNR Istituto di Scienze e Tecnologie Chimiche ‘‘Giulio Natta’’, Sede Via C. Golgi, 19, 20133 Milano, Italy

b) Istituto Lombardo, Accademia di Scienze e Lettere, via Brera 76, 20121 Milano, Italy

c) Institut des Sciences Moléculaires, UMR CNRS 5255, 351 Cours de la Libération, 33405 Talence, France

d) Laboratoire de Réactivité et Chimie des Solides (LRCS), UMR CNRS 7314, Université de Picardie Jules Verne, Hub de l’Energie, 15 Rue Baudelocque, 80000 Amiens Cedex, France. Réseau sur le Stockage Electrochimique de l’Energie (RS2E), FR CNRS 3459, France.

(*) ORCID 0000-0002-0047-1596; e-mail c.gatti@scitec.cnr.it

Table S1 Values (in Debye, D) of the elements of the matrices $M^{x,+}$ and $M^{x,-}$ relative to the $S_1 \leftarrow S_0$ transition in the systems: BM+ES_2 in water and BM in CCl_4 . The elements of the matrices of the components *y* and *z* of the $\boldsymbol{\mu}_{CT}$ vector have values that are, in general, comparatively much smaller in magnitude than those of the *x* component matrix. The sum of the elements on the diagonal of the matrix $M^{x,+}$ (or $M^{x,-}$) (those with pink background) corresponds to the *intra*-subdomains contribution, μ_{CT}^{intra}, to $\boldsymbol{\mu}_{CT,x}^{+}$ (or to $\boldsymbol{\mu}_{CT,x}^{-}$) while the sum of the out of diagonal elements (those with light blue background) corresponds to the *inter*-subdomains contribution, μ_{CT}^{inter}, to $\boldsymbol{\mu}_{CT,x}^{+}$ (or to $\boldsymbol{\mu}_{CT,x}^{-}$) (Eq. 15).

	$\boldsymbol{M}^{x,+}$ *matrix* for $S_1 \leftarrow S_0$ of BM + ES_2 in water				$\boldsymbol{M}^{x,+}$ *matrix* for $S_1 \leftarrow S_0$ of BM in CCl_4			
	$q_{CT}^{+}(\mathrm{O(CO)})$	$q_{CT}^{+}(\mathrm{6CMR})$	$q_{CT}^{+}(\mathrm{CB})$	$q_{CT}^{+}(\mathrm{MePy})$	$q_{CT}^{+}(\mathrm{O(CO)})$	$q_{CT}^{+}(\mathrm{6CMR})$	$q_{CT}^{+}(\mathrm{CB})$	$q_{CT}^{+}(\mathrm{MePy})$
$\mathbf{d}_{CT,x}^{\mathrm{O(CO)}}$	*-0.483*	-1.400	-0.480	-0.346	*0.003*	0.058	0.066	0.053
$\mathbf{d}_{CT,x}^{\mathrm{6CMR}}$	-0.536	*-1.561*	-0.536	-0.384	0.003	*0.061*	0.069	0.058
$\mathbf{d}_{CT,x}^{CB}$	0.036	0.102	*0.036*	0.025	0.005	0.114	*0.130*	0.107
$\mathbf{d}_{CT,x}^{MePy}$	-1.324	-3.843	-1.319	*-0.946*	-0.000	-0.020	-0.023	*-0.018*
	$\boldsymbol{M}^{x,-}$ *matrix* for $S_1 \leftarrow S_0$ of BM + ES_2 in water				$\boldsymbol{M}^{x,-}$ *matrix* for $S_1 \leftarrow S_0$ of BM in CCl_4			
	$q_{CT}^{-}(\mathrm{O(CO)})$	$q_{CT}^{-}(\mathrm{6CMR})$	$q_{CT}^{-}(\mathrm{CB})$	$q_{CT}^{-}(\mathrm{MePy})$	$q_{CT}^{-}(\mathrm{O(CO)})$	$q_{CT}^{-}(\mathrm{6CMR})$	$q_{CT}^{-}(\mathrm{CB})$	$q_{CT}^{-}(\mathrm{MePy})$
$\mathbf{d}_{CT,x}^{\mathrm{O(CO)}}$	*-0.071*	-0.368	-0.613	-1.660	*0.005*	0.058	0.058	0.058
$\mathbf{d}_{CT,x}^{\mathrm{6CMR}}$	-0.081	*-0.409*	-0.679	-1.848	0.005	*0.061*	0.064	0.061
$\mathbf{d}_{CT,x}^{CB}$	0.005	0.025	*0.043*	0.119	0.010	0.114	*0.117*	0.114
$\mathbf{d}_{CT,x}^{MePy}$	-0.198	-1.012	-1.675	*-4.552*	-0.003	-0.020	-0.020	*-0.020*

Table S2 Values (in Debye, D) of the elements of the matrices $M^{x,+}$ and $M^{x,-}$ relative to the $S_2 \leftarrow S_0$ transition in the systems: BM+ES_2 in water and BM in CCl_4 . The elements of the matrices of the components *y* and *z* of the $\boldsymbol{\mu}_{CT}$ vector have values that are, in general, comparatively much smaller in magnitude than those of the *x* component matrix. The sum of the elements on the diagonal of the matrix $M^{x,+}$ (or $M^{x,-}$) (those with pink background) corresponds to the *intra*-subdomains contribution, μ_{CT}^{intra}, to $\boldsymbol{\mu}_{CT,x}^{+}$ (or to $\boldsymbol{\mu}_{CT,x}^{-}$) while the sum of the out of diagonal elements (those with light blue background) corresponds to the *inter*-subdomains contribution, μ_{CT}^{inter}, to $\boldsymbol{\mu}_{CT,x}^{+}$ (or to $\boldsymbol{\mu}_{CT,x}^{-}$) (Eq. 15).

	$M^{x,+}$ *matrix* for $S_2 \leftarrow S_0$ of BM + ES_2 in water				$M^{x,+}$ *matrix* for $S_2 \leftarrow S_0$ of BM in CCl_4			
	$q_{CT}^{+}(\mathrm{O(CO)})$	$q_{CT}^{+}(\mathrm{6CMR})$	$q_{CT}^{+}(\mathrm{CB})$	$q_{CT}^{+}(\mathrm{MePy})$	$q_{CT}^{+}(\mathrm{O(CO)})$	$q_{CT}^{+}(\mathrm{6CMR})$	$q_{CT}^{+}(\mathrm{CB})$	$q_{CT}^{+}(\mathrm{MePy})$
$\mathbf{d}_{CT,x}^{\mathrm{O(CO)}}$	*-0.696*	-1.334	-0.709	-0.646	*-4.931*	-2.511	-0.351	-0.397
$\mathbf{d}_{CT,x}^{\mathrm{6CMR}}$	-0.529	*-1.009*	-0.539	-0.488	0.702	*0.356*	0.051	0.056
$\mathbf{d}_{CT,x}^{CB}$	0.163	0.310	*0.165*	0.150	0.127	0.064	*0.010*	0.010
$\mathbf{d}_{CT,x}^{MePy}$	-2.031	-3.881	-2.066	*-1.876*	-1.721	-0.877	-0.122	*-0.137*
	$M^{x,-}$ *matrix* for $S_2 \leftarrow S_0$ of BM + ES_2 in water				$M^{x,-}$ *matrix* for $S_2 \leftarrow S_0$ of BM in CCl_4			
	$q_{CT}^{-}(\mathrm{O(CO)})$	$q_{CT}^{-}(\mathrm{6CMR})$	$q_{CT}^{-}(\mathrm{CB})$	$q_{CT}^{-}(\mathrm{MePy})$	$q_{CT}^{-}(\mathrm{O(CO)})$	$q_{CT}^{-}(\mathrm{6CMR})$	$q_{CT}^{-}(\mathrm{CB})$	$q_{CT}^{-}(\mathrm{MePy})$
$\mathbf{d}_{CT,x}^{\mathrm{O(CO)}}$	*-0.112*	-0.310	-0.206	-2.760	*-2.257*	-2.837	-1.721	-1.977
$\mathbf{d}_{CT,x}^{\mathrm{6CMR}}$	-0.084	*-0.236*	-0.155	-2.089	0.320	*0.404*	0.132	0.282
$\mathbf{d}_{CT,x}^{CB}$	0.025	0.072	*0.048*	0.641	0.058	0.074	*0.028*	0.051
$\mathbf{d}_{CT,x}^{MePy}$	-0.325	-0.905	-0.597	*-8.032*	-0.788	-0.991	-0.389	*-0.691*

Table S3 Values (in Debye, D) of the elements of the matrices $M^{x,+}$ and $M^{x,-}$ relative to the $S_3 \leftarrow S_0$ transition in the systems: BM+ES_2 in water and BM in CCl_4 . The elements of the matrices of the components *y* and *z* of the $\boldsymbol{\mu}_{CT}$ vector have values that are, in general, comparatively much smaller in magnitude than those of the *x* component matrix. The sum of the elements on the diagonal of the matrix $M^{x,+}$ (or $M^{x,-}$) (those with pink background) corresponds to the *intra*-subdomains contribution, μ_{CT}^{intra}, to $\boldsymbol{\mu}_{CT,x}^{+}$ (or to $\boldsymbol{\mu}_{CT,x}^{-}$) while the sum of the out of diagonal elements (those with light blue background) corresponds to the *inter*-subdomains contribution, μ_{CT}^{inter}, to $\boldsymbol{\mu}_{CT,x}^{+}$ (or to $\boldsymbol{\mu}_{CT,x}^{-}$) (Eq. 15).

	**$M^{x,+}$ *matrix* for $S_3 \leftarrow S_0$ of BM + ES_2 in water**				**$M^{x,+}$ *matrix* for $S_3 \leftarrow S_0$ of BM in CCl_4**			
	q_{CT}^{+}(O(CO))	q_{CT}^{+}(6CMR)	q_{CT}^{+}(CB)	q_{CT}^{+}(MePy)	q_{CT}^{+}(O(CO))	q_{CT}^{+}(6CMR)	q_{CT}^{+}(CB)	q_{CT}^{+}(MeyY)
$\mathbf{d}_{CT,x}^{O(CO)}$	*-7.269*	-3.276	-0.465	-0.834	*-0.119*	-0.493	-0.356	-0.607
$\mathbf{d}_{CT,x}^{6CMR}$	-0.338	*-0.153*	-0.020	-0.038	-0.239	*-0.994*	-0.719	-1.225
$\mathbf{d}_{CT,x}^{CB}$	-0.407	-0.183	*-0.025*	-0.046	0.023	0.097	*0.071*	0.119
$\mathbf{d}_{CT,x}^{MePy}$	-4.784	-2.155	-0.305	*-0.549*	-0.419	-1.749	-1.268	*-2.155*
	**$M^{x,-}$ *matrix* for $S_3 \leftarrow S_0$ of BM + ES_2 in water**				**$M^{x,-}$ *matrix* for $S_3 \leftarrow S_0$ of BM in CCl_4**			
	q_{CT}^{-}(O(CO))	q_{CT}^{-}(6CMR)	q_{CT}^{-}(CB)	q_{CT}^{-}(MePy)	q_{CT}^{-}(O(CO))	q_{CT}^{-}(6CMR)	q_{CT}^{-}(CB)	q_{CT}^{-}(MePy)
$\mathbf{d}_{CT,x}^{O(CO)}$	*-1.845*	-2.783	-2.346	-4.931	*-0.018*	-0.097	-0.104	-1.355
$\mathbf{d}_{CT,x}^{6CMR}$	-0.086	*-0.130*	-0.109	-0.229	-0.033	*-0.198*	*-0.214*	-2.732
$\mathbf{d}_{CT,x}^{CB}$	-0.104	-0.155	*-0.132*	-0.275	0.003	0.020	*0.020*	0.267
$\mathbf{d}_{CT,x}^{MePy}$	-1.215	-1.833	-1.545	*-3.246*	-0.058	-0.346	-0.374	*-4.812*
